# Design Method and Teeth Contact Simulation of PEEK Involute Spline Couplings

**DOI:** 10.3390/ma17010060

**Published:** 2023-12-22

**Authors:** Xiangzhen Xue, Wei Yu, Kuan Lin, Ning Zhang, Li Xiao, Yiqiang Jiang

**Affiliations:** 1 School of Mechanical and Electrical Engineering, Shaanxi University of Science and Technology, Xi’an 710021, China; 220512054@sust.edu.cn (W.Y.); 220512094@sust.edu.cn (K.L.); 220511005@sust.edu.cn (N.Z.); xiaoli@sust.edu.cn (L.X.); 2 Department of International Applied Technology, Yibin University, No. 8. Luke, Wuliangye Avenue, Yibin 644002, China; 210512100@sust.edu.cn

**Keywords:** contact characteristic analysis, involute splines design, material viscoelasticity, optimized design, PEEK (polyetheretherketone)

## Abstract

In order to design an involute spline made of PEEK (polyetheretherketone)-based material with better performance and improve the design rules of involute splines, initially, an involute splines design theory for PEEK (polyetheretherketone)-based materials is presented, which combines international standards (ISO 4156, 1. 2. 3, 2005), American standards (ANSI B92.2M. 1989), and traditional empirical formulas. Second, using the involute splines calibration method in international standards as a guide, we developed the involute splines calibration method for PEEK-based materials by estimating the impact of energy consumption caused by viscoelasticity on temperature field calibration. Next, the contact characteristics of the designed spline were analyzed using ABAQUS2022 software to confirm the accuracy and reliability of the design and calibration methods. Finally, finite element simulation was used to analyze the influence of different pressure angles, moduli, combined lengths, and other parameters on the contact characteristics of the spline in order to realize the optimal design of PEEK-based material involute splines, to offer a theoretical foundation and improved design methodology for cylindrical straight-tooth involute splines.

## 1. Introduction

Involute splines are significant power transmission components in mechanical transmission systems because of their excellent mechanical strength. They are widely used in aircrafts, automotives, heavy machinery, and construction machinery. With the emergence of high-performance drive systems, the demand for involute splines that are dependable, safe, and have long lifespans has increased. Due to these stringent criteria, the impact of difficult working circumstances, and the constraints of its own geometric construction, spline vice wear and tear is an increasingly serious concern and is prone to disastrous incidents [1,2,3]. During the development of PEEK-based materials and lightweight involute splines using advanced manufacturing methods for driving training and design goals, we should achieve reliable transmission systems to reduce wear and abrasion and meet the global requirements of green development. PEEK is a high-performance heat-resistant polymer that was developed by ICI in the early 1980s. It exhibits exceptional self-lubricating wear resistance and fatigue resistance, and these qualities can be modified to achieve good dimensional stability and rigidity. It has been widely used to manufacture gears, bushings, and bearing parts for light-load and low-speed applications in the fields of aviation, aerospace, and robotics [4]. Implementing such splines in the future is crucial for investigating the design theory of involute splines in PEEK-based materials and their contact characteristics.

Contemporary research has focused on enhancing the overall performance of involute splines and weakening the impact of micro-motion wear. Leen et al. validated finite element analyses by studying splines under various situations using the finite element approach and obtained the stress distribution on spline tooth surfaces [5,6,7]. Patil et al. used the finite element approach to examine the load distribution of splines and the frictional contact and load carrying capacities [8,9]. Volfson et al. developed a contact model for splines and assessed their contact features; they presented stress parameters that influenced the lifespan of spline subs [10,11,12]. Lyashuk et al. used finite element modeling and adjusted spline subdimensional parameters to increase the ability of the transmission system to carry higher loads [13,14]. Stenstrand et al. developed a mathematical model for spline subdesign after studying the automated spline subdesign process [15,16]. Han Zhengbiao et al. investigated the contact properties of the drive involute splines and compared the influence of different circumstances on the splines [8,9,17]. Yu Sitai et al. established a mathematical model based on fretting wear and analyzed the influence of fretting wear. [18,19,20,21]. Ting-Yue Wang et al. investigated the contact stiffness of spline connection structures and the vibration characteristics of the rotor systems and then analyzed the inherent characteristics of the spline [22,23]. Faidh-Allah et al. used the finite element method to conduct numerical research on the induced design parameters of the internal and external spline shafts and compared them through experiments to obtain the stress variation rule. [24,25]. The aforementioned research has provided a robust foundation for investigating the contact properties of involute splines pairs fabricated using PEEK-based materials; researchers have presented excellent results in the fields of splines design, dynamic properties, frictional wear, and fatigue life. Contemporary studies on involute splines are based on splines made of metallic materials, and only a few studies have designed and examined the contact characteristics of composite spline subsets. There are still some shortcomings in the current research, including insufficient in-depth research on the contact characteristics of PEEK-based material involute spline coupling, insufficient theoretical analysis and mechanism interpretation, a lack of uniformity of test methods and evaluation standards, and insufficient research on long-term performance and durability. In view of these problems, future research needs to strengthen the combination of theory and experiment, provide comprehensive and accurate data and analysis, and provide a more reliable scientific basis for the design and application of PEEK-based materials that involve spline couplings.

In this study, based on the existing standards and traditional empirical formula [26], combined with the parameter design of metal involute splines and the strength checking theory, this paper synthesized the viscoelasticity, energy consumption, and temperature field effects of PEEK-based polymer composites. The design theory of an involute spline of PEEK (polyetheretherketone)-based material is presented. A finite element model was developed using the PEEK-based involute splines, and it was used to analyze the contact characteristics of the involute splines pair in terms of tooth contact pressure distribution, inter-tooth load distribution, and maximum contact stress. The effects of various pressure angles, moduli, and engagement length on the contact properties of the as-developed involute splines vice was investigated. The findings of this study provide significant insights for designing lightweight and high-performance involute splines vices with high precision, reliability, and strengths.

## 2. Involute Splines Design Theory for PEEK-Based Materials

Involute splines are designed using metallic materials following international, American, German, British, and French standards. These standards specify the design, dimensions, and load capacity calculations of involute splines subparameters. To the best of our knowledge, this is the first study to propose a method for designing involute splines subsets using PEEK-based materials. In composite materials, anisotropy is caused by the arrangement of fibers in different directions inside the matrix. Different material properties of fibers and matrix phases result in different material properties; fiber orientation thus depends on the type and volume fraction of the component materials as well as on the distribution patterns of the reinforcing fibers. To reasonably design involute splines using PEEK-based materials, the design method and process of involute splines based on metallic materials should be considered, along with economically feasible solutions for meeting the design requirements, contemporary processes, and the possible superposition effect caused by the addition of various components during practical applications. In this study, the design for preparing involute splines vice using metallic materials and the international standard “ISO 4156. 1. 2. 3-2005” [27] are combined with parameter design, and the method and parameters for preparing involute splines using PEEK-based materials are proposed. Considering the anisotropic and viscoelastic properties of PEEK-based materials, checks were performed for strength, wear resistance, and tooth temperature along with the international standard and “ANSI AGMA 6123-C16”, [28] obtaining PEEK-based material involute splines vices with high accuracy, reliability, and long life.

### 2.1. Involute Splines Parameter Design

#### 2.1.1. Basic Tooth Profile and Pressure Angle Determination

The standard “ISO 4156. 1. 2. 3-2005” was used to determine the dimensions of involute splines. Three pressure angles (denoted by *α*) and two tooth roots, namely, a 30° flat tooth root, a 30° round tooth root, a 37.5° round tooth root, and a 45° round tooth root, were used to specify the four basic tooth profiles of cylindrical straight-tooth involute splines. For the PEEK-based material, four tooth profiles and three pressure angles are selected. Except for the modulus *m* = 1.0, the pressure angle on the reference circle is 30° for spline connections of other modules. The fit tolerance of the involute splines is H/h. The precise choice was considering the working circumstances.

#### 2.1.2. Selection of Modulus

Several essential standards for splines adopt the pitch diameter system, that is, in addition to the number of teeth, the pitch, *p*, is one of the primary factors that influence the properties of involute splines. Standards such as French and American ones use essentially the same modulo scheme as the international standard, and an increasing number of standards are adopting the same. The modulus is defined as a basic parameter of the involute splines to measure the size of the gear teeth. The modulus is an important geometric parameter influencing the size of involute splines key teeth. Its value (in mm) is obtained by dividing the tooth pitch by π. Similar to gears, the modulus of involute splines is divided into two series, with the first series being preferred, and it is 0.25, 0.50, 1, 1.5, 2, 2.5, 3, 5, and 10.

The strength of an involute spline is typically correlated with its diameter rather than its modulus. The number of teeth considered is higher and the modulus is smaller.

#### 2.1.3. Determination of the Engagement Length

The torsional strength of a shaft and the shear strength of spline teeth, among other factors, determine the involute splines’ engagement length, *l*. Considering that the dangerous section of an involute spline is at the mean circular diameter, *D_m_*, of the spline connection, *l* was calculated using Equation (1) [29]:

(1) $$l=\frac{\left(D_{B}^{4}-D_{l}^{4}\right)}{D_{m}^{2}D_{B}}$$

*l* should not be too large because the excess bonding length can lead to uneven distribution of load along the spline vice axis. Increasing the length does not increase the load carrying capacity; in the case of transferring torque only, the general initial design takes “$l=0.5D_{m}$” [29].

For involute splines made of PEEK-based materials, the initial engagement length was determined using Equation (1). Based on this value, the contact characteristics of splines of this material with different engagement lengths were analyzed using the Abaqus simulation, and the methods and principles of the bond length of involute splines of PEEK-based materials were obtained.

#### 2.1.4. Determination of the Number of Teeth

For determining the number of teeth, according to the American Standard, we considered number of teeth in the range 6–60 at a pressure angle of 30°–37.5° and 6–100 at a pressure angle of 45°. According to the American Standard, it is best to have an even number of involute splines teeth, especially for internal splines; if the number of teeth is odd, it is considered unfavorable to the measurement.

The general simple formulas [26] for calculating the strength of involute splines connections are given using Equations (2)–(4):

(2) $$p=\frac{2T\times 10^{3}}{\psi zhld_{m}}\leq \left[p\right]$$

The input torque *T* is:

(3) $$T=9550\frac{P}{n}$$

**Figure 1:**
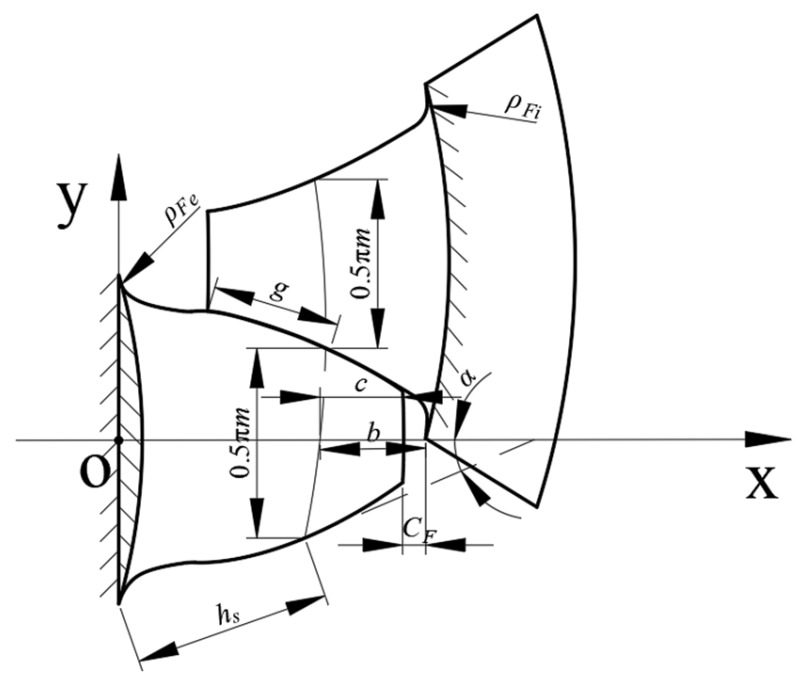
Schematic diagram of key teeth parameters.

The average circle diameter *D_m_* is:

*D_m_* = *D* = *mz* (4)

where *h* is the working height of the key teeth (mm). According to Figure 1, if the working height of the key teeth cannot be determined by the following chart, it takes *h* = *m* (*α* = 30°) [28].

Permissible stress [*p*] was determined using Equation (5):

(5) $$\left[p\right]=\frac{\sigma^{o}}{s}$$

where *σ^0^* is the ultimate material stress. For plastic materials, the yield limit of the material *σ_s_* as its design material ultimate stress *σ^0^* [30].

At a known input torque *T* and modulus *m*, the number of teeth *z* was calculated using Equation (6). For example, for 30° circular root, the number of teeth of the spline at *α_D_* = 30° was:

(6) $$z=\sqrt[{3}]{\frac{1000\times T}{0.1875pm^{3}}}$$

*z* for meeting the minimum number of teeth condition [31]:

(7) $$z_{min}\geq \frac{D_{B}}{m}+2h_{f}^{*}$$

which is given using Equation (8):

(8) $$D_{B}=0.8D$$

where $h_{f}^{*}$ is the tooth root height factor, taken as 0.7 [31].

#### 2.1.5. Selection of Centering Method

The centering method was selected after designing and calculating the basic geometric parameters of the splines. While tooth side centering is used typically, when the radial force is relatively large, the method selected for tooth side centering caused significant eccentricity between the inner and outer splines. Moreover, the force between the teeth was distributed in fewer pairs of teeth, which increased the pressure on the tooth surface and accelerated key tooth wear; thus, large- or small-diameter centering was used to spread the radial force across the centering mating surface, which improved the tooth wear.

In case of large or small diameter centering, sufficient tooth side clearance was ensured to avoid overpositioning. Table 5 of the German standard “DIN5480-1” [32] gives the recommended tolerance zone for the diameter of the tip circle and the diameter of the root circle, in this case, the corresponding tooth side fit.

#### 2.1.6. Analysis of the Calculation Results of Basic Parameters

We considered PEEK5600CF30 material involute splines as an example and simulated the actual working conditions and designed the involute splines according to the design steps proposed herein. The input power and speed were *p* = 1100 (KW) and *n* = 6000 (rpm), respectively. Material PEEK5600CF30 was selected, and its related mechanical properties and indicators are presented in Appendix B. After strength checks, if the calculated number of teeth did not satisfy the strength requirements, the number of teeth was increased accordingly. More teeth in the American standard implied better quality; the number of teeth increased in the range of 8–65 (8, 10, 11, 12, in increments of 2, up to 65). The preliminary calculations of the basic dimensions of the PEEK-based involute splines are presented in Table 1.

All other geometrical parameters, with the exception of the spline-specific geometrical parameters shown in Table 2, were calculated according to the international standard “ISO 4156, 1. 2. 3, 2005” dimensional series parameter calculation method. Symbols used in this study were also consistent with this standard. The geometric parameter relationships and fit tolerances specified in the standard were maintained in the calculation.

### 2.2. Involute Splines Strength Checking

Involute splines strength calculation included tooth surface contact strength analysis, tooth root bending strength analysis, tooth root shear strength calculation, external spline torsion, bending strength calculation, and so on. The specific process of these calibrations was according to “ISO 4156. 1. 2. 3-2005”. Due to the unique properties of PEEK, which are different from those of metallic materials in some specific aspects, further pertinent strength calculations were needed to enable improved dependability and longer service life.

#### 2.2.1. Shear Strength Check of Internal Splines

Shear strength calibration was carried out for involute splines fabricated using the PEEK-based material. For solid shafts, if the coupling teeth were less than one-third of the pitch diameter and the minimum diameter of the shaft was the root diameter of the coupling, the shear capacity of the shaft exceeded the shear capacity of the teeth, i.e., the maximum shear strength of the shaft was greater than the shear strength of the teeth. Before calibrating this strength, the break-away stress, *S*_1_, caused by the radial component of the diagonal force was calculated, drawing bending stress, *S*_2_, and the circumferential stress caused by circumferential stress, *S*_3_, as follows:

(9) $$S_{1}=\frac{1000T\tan\alpha_{n}}{\pi dt_{w}l}$$

The wall thickness of the splines is:

(10) $$t_{w}=0.5\pi m$$

Drawing bending stress was:

(11) $$S_{2}=\frac{4000T}{d^{2}lY}$$

Note: In this equation, we assumed that the load was carried on half of the spline.

The circumferential stress caused by circumferential stress was:

(12) $$S_{3}=8.85\times 10^{-12}d_{x}{}^{2}(d_{so}^{2}+0.424d_{n}^{2})$$

Input power was:

(13) P=Tω

The coupling speed was:

(14) $$d_{so}=\omega \times R$$

The final total tensile stress was:

(15) $$S_{t}=K_{m}\left(S_{1}+S_{2}\right)+S_{3}$$

If $S_{t}\leq S_{tA}$, then the shear strength of the inner spline was designed to meet the requirements, where $S_{tA}$ is the permissible stress; $H_{c\;}$ is the core hardness; *C* is Rockwell hardness; $K_{m}$ is load distribution factor [33].

#### 2.2.2. Other Strength Calculations

(1) Calculation of wear resistance when operating at 10^8^ cycles:

Under normal circumstances, the composite involute splines were easily fractured due to tooth wear. Since this phenomenon is more likely to occur under conditions of high load and poor heat dissipation, the strength was designed and calculated using the face pressure of Hertzian stress. Wear resistance was calculated according to the calculation method in “ISO4156. 1. 2. 3—2005” using Equations (16) and (17):

(16) $$S_{c}=\sqrt{\frac{2F_{t}}{Bd1}\;}\times \sqrt{\frac{1.4}{\left(\frac{1}{E_{1}}+\frac{1}{E_{2}}\right)sin2a}}$$

*B* = 0.5π*m* (17)

The face pressure of the composite involute splines during operation was calculated and compared with the allowable compressive stress to obtain its compliance with the regulations.

Known allowable compressive stress of tooth surface $\sigma_{Hp}$, if $S_{c}<\sigma_{Hp}$ conformed to design requirements.

We calculated $\sigma_{H}$ and allowable compressive stress of tooth surface $\sigma_{Hp1}$ [34]. If $\sigma_{H}<\sigma_{Hp1}$, the specimen conformed to design requirements.

(2) Calculation of wear resistance during long-term operation:

*HRC =* 6.96 + 0.089*HB* (18)

where *HRC* is the material Rockwell hardness, which can be obtained from Appendix B; *HB* is Brinell hardness; substitution parameters can be obtained for Brinell hardness.

Allowable compressive stress for tooth wear “$\sigma_{Hp2}=0.028\times HB$” [34].

After calculation, if the strength condition of $\sigma_{H}\leq \sigma_{Hp2}$ was satisfied, the specimen was said to have a long lifetime.

#### 2.2.3. Allowable Torque Calibration

The calibration of permissible torque was based on ISO 4156.1. 2. 3-2005 and was derived from the inverse of the tooth surface permissible compressive stress calibration, mainly using the tooth surface permissible compressive stress as the starting point.

Allowable compressive stress of the tooth surface:

(19) $$\sigma_{Hp}=\sigma_{0.2}/\left(S_{H}K_{1}K_{2}K_{3}K_{4}\right)$$

Working tooth height:

(20) $$h_{w}=\left(D_{ee}-D_{ii}\right)/2$$

The allowable stress:

(21) $$\sigma_{H}=\frac{\left[W\right]}{h_{w}}$$

Permitted unit loads:

(22) $$\left[W\right]=\frac{\left[F_{t}\right]}{\left(zlcos\alpha_{D}\right)}$$

The permissible circumferential force:

(23) $$\left[F_{t}\right]=\frac{2000T}{D}$$

PEEK polymer composite involute spline couplings allowed a torque of:

(24) $$\left[T\right]=\left[F_{t}\right]\frac{D}{2000}$$

Maximum torque of the involute splines:

(25) $$T_{a}=9550\frac{P}{n}$$

The permissible torque was greater than the maximum torque at the coupling; *T_a_* < [*T*] was in accordance with the design requirements. The above torque check was for the middle position of the teeth

### 2.3. Temperature Field Calibration

Due to the inherent inhomogeneity of the composite material, its anisotropy, designability, and viscoelasticity of the polymer conforming to the material, among other characteristics, temperature is one of the important parameters affecting the working performance of the PEEK-based involute splines. When the PEEK-based involute splines were engaged, the frictional heat of the tooth surface and the unique internal heat dissipation of the viscoelastic material were not rapidly transferred through the key tooth material, resulting in poor thermal conductivity and increasing the temperature of the key tooth. The loss of energy also generated some heat, which also resulted in changes in tooth shape. A very high local temperature increase occurred in the form of tooth surface burns or melting and other forms of damage different from the metal spline. As a result, heat generation calculations are crucial for designing material-compliant involute splines.

#### Stress-Strain Relationships for PEEK-Based Materials

**Figure 2:**
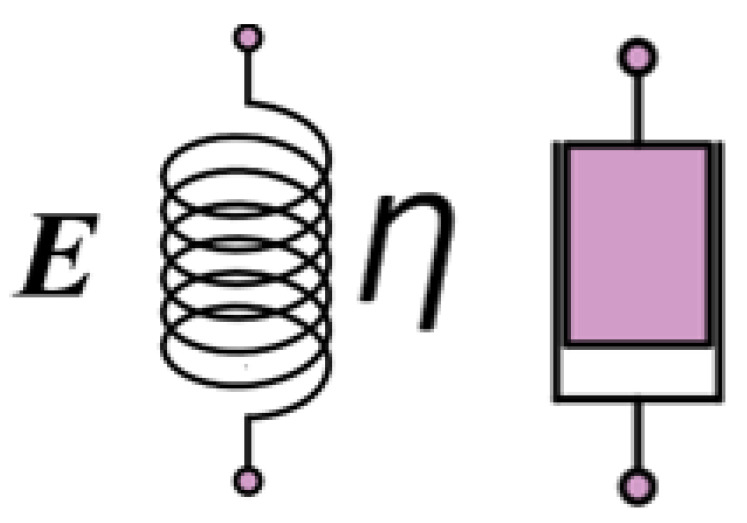
Spring damping diagram.

As show in Figure 2, the stress–strain relationship for metallic materials at room temperature is depicted by Hooke’s law, which gives an ideal elastic model that can be represented by a spring:

(26) σ=Eε

The ideal viscous model was a dampener that provided positive proportionality between stress and strain rate:

(27) $$\sigma =\eta \dot{\varepsilon }$$

where *η* is the Coefficient of viscosity; $\dot{\varepsilon }$ is the Strain rate.

**Figure 3:**
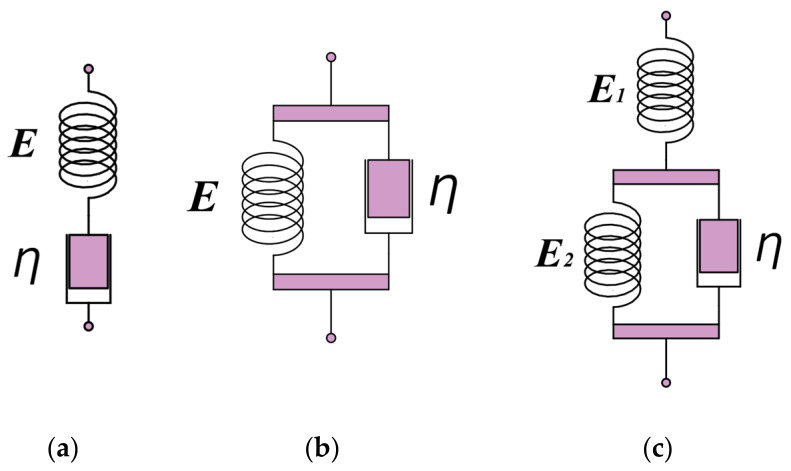
Diagram of viscoelastic model. (**a**) Maxwell models, (**b**) Voigt models, (**c**) three elements model.

A spring and a damper were combined to form a variety of viscoelastic models, such as a Maxwell model with the two in series, a Voigt model with the two in parallel, and a Voigt model with a spring in series to form a three elements model, as show in Figure 3. One of the three elements model was more applicable to plastic materials, and it provided the differential stress–strain relationship [35]:

(28) $$\frac{E_{2}\eta }{E_{1}+E_{2}}\dot{\varepsilon }+\frac{E_{1}E_{2}}{E_{1}+E_{2}}\varepsilon =\sigma +\frac{\eta }{E_{1}+E_{2}}\dot{\sigma }$$

The viscoelastic phenomenon was described qualitatively and macroscopically by various combinations of springs and dampers.

The mechanical properties of a material made under alternating stress or alternating strain are called “dynamic mechanical behavior”, which is often an expression of the relationship between modulus, internal dissipation, frequency, and temperature. The viscoelastic behavior during the vibration of the object is called dynamic viscoelasticity. To study the viscoelastic behavior of materials under dynamic loading, it is necessary to determine the response of viscoelastic materials under alternating stress or alternating strain and investigate the expression of the physical quantities related to dynamic viscoelastic properties.

During the actual operation of an involute splines, the surface of the material was subjected to external alternating forces. We assumed the following complex expression of the vibrational strain on the material:

(29) $$\begin{array}{ll} \varepsilon \left(t\right) & =\varepsilon_{0}e^{i\omega t}\\ & =\varepsilon_{0}(\cos\omega t+\mathrm{isin}\omega t) \end{array}$$

where $\varepsilon_{0}$ is the Strain amplitude; *ω* is the change in the angle of rotation around the axis per unit time.

The strain expression was split into real and imaginary halves at every time *t*, where the real part was represented by $\varepsilon_{0}$ cos*ωt* and the imaginary part by $\varepsilon_{0}$sin*ωt*.

The general expression for the linear viscoelastic differential type constitutive equation for viscoelastic materials was [34]:

(30) $$\sum_{k=0}^{n}\left(P_{k}\frac{d^{k}\varepsilon }{dt^{k}}\right)=\sum_{k=0}^{m}\left(Q_{k}\frac{d^{k}\sigma }{dt^{k}}\right)(n\geq m)$$

It was reduced to:

(31) Pσ=Qε

where $P_{k}$ and $Q_{k}$ are the constants of the material’s properties.

**Figure 4:**
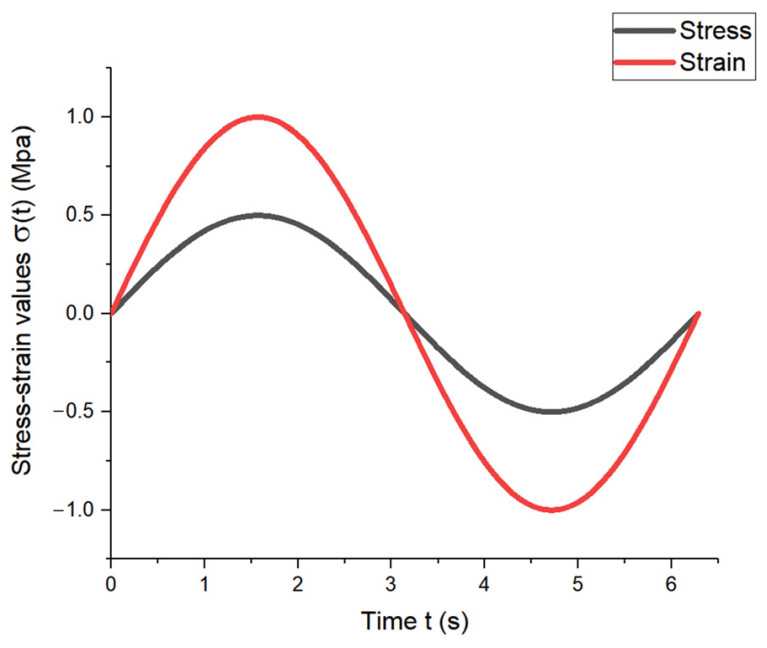
Stress–strain variation diagram of elastic material.

When an elastic material was subjected to sine or cosine stress, the strain and stress changed with the same phase position and amplitude value in the same direction as the sine or cosine, as shown in Figure 4.

**Figure 5:**
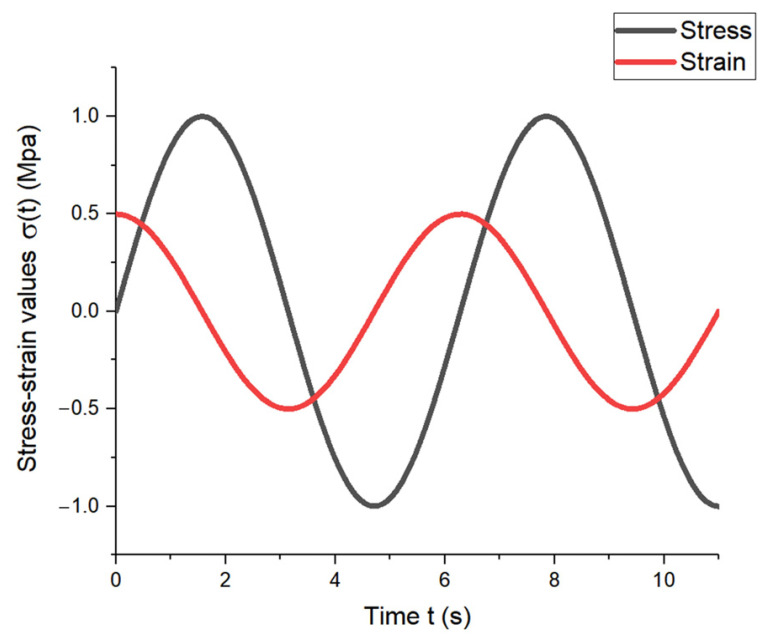
Stress–strain variation diagram of viscous material.

When a viscous material is subjected to a sine or cosine stress, the strain and stress change in the same direction but at different phases, resulting in a strain lag time of π/2*ω*, as illustrated in Figure 5.

**Figure 6:**
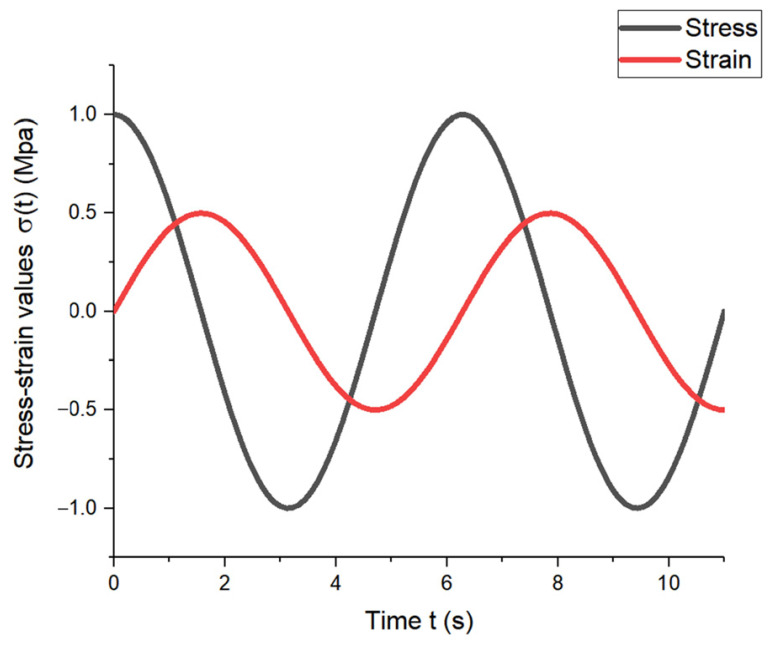
Stress–strain variation diagram of viscoelastic material.

For viscoelastic materials, the stress–strain response is between the above two, and the phase difference of the lag is expressed by *δ*, with 0 < *δ* < π/2*ω*, as shown in Figure 6.

Therefore, for PEEK-based materials, the stress–strain response lagged behind a phase difference *δ*. The stress response also alternated, and the complex expression for the vibrational stress was:

(32) $$\sigma \left(t\right)=\sigma_{0}e^{i(\omega t+\delta )}$$

By bringing the expression ε(t), σ(t) into the general expression of the linear viscoelastic differential type intrinsic constitutive equation, we obtained the relationship between stress and strain.

(33) $$\sigma \left(t\right)\;=\;Y^{*}\varepsilon \left(t\right)$$

where $Y^{*}$ is a complicated function expression, representing a function of frequency that does not vary with time and is often referred to as a composite modulus. Its complicated function is $Y^{*}=Y_{1}+Y_{2}I$.

An elastomer and a liquid combine to form a viscoelastic body. A specific viscosity was produced inside the viscoelastic material when it was subjected to alternating loads. Viscosity, according to the law of Newton inner friction, is explained as the effect of molecular diffusion or mutual attraction between molecules, which results in momentum exchange between fluids with different flow rates. Internal friction was generated on the contact surface of the two flow layers inside the fluid, which eventually generated some heat inside it, resulting in energy consumption.

The loss of energy consumption included both elastic potential energy and dissipative energy, and the energy consumption of PEEK-based materials was determined by first calculating the energy per unit volume [35]:

(34) $$W=\int^{\;}\sigma \left(t\right)d\varepsilon =\int^{\;}\sigma \left(t\right)\dot{\varepsilon \left(t\right)}dt$$

The derivative of the function ε(t) with respect to time *t* yielded:

(35) $$\dot{\varepsilon \left(t\right)}=\varepsilon_{0}i\omega e^{i\omega t}=i\omega \varepsilon \left(t\right)$$

We obtained:

(36) $$\varepsilon \left(t\right)=\frac{\dot{\varepsilon \left(t\right)}}{i\omega }$$

ε(t) was inserted in Equation (36) to obtain the relationship between stress and strain:

(37) $$\sigma \left(t\right)\;=Y_{1}\varepsilon \left(t\right)+\frac{Y_{2}}{\omega }\dot{\varepsilon \left(t\right)}$$

Equation (37) was inserted in the expression for energy consumption and integrated over period T to obtain Equation (38).

(38) $$\begin{array}{ll} W & =\int_{0}^{T}\sigma \left(t\right)\dot{\varepsilon \left(t\right)}dt\\ & =\int_{0}^{T}Y_{1}\varepsilon \left(t\right)d\varepsilon \left(t\right)+\int_{0}^{T}\frac{Y_{2}}{\omega }{\dot{\varepsilon \left(t\right)}}^{2}dt\\ & =\frac{1}{2}Y_{1}\varepsilon {\left(t\right)}^{2}|\begin{array}{c} T\\ 0 \end{array}+\int_{0}^{T}\frac{Y_{2}}{\omega }{\dot{\varepsilon \left(t\right)}}^{2}dt \end{array}$$

Equation (38) represents the work done per unit volume in one cycle. The first term in the equation was elastic potential energy, which was reversible, while the second term represented the energy loss due to viscosity, which was irreversible. The real and imaginary parts $Y_{1}$ and $Y_{2}$ of the composite modulus were the storage and loss modulus, respectively. The energy $W_{d}$ lost per unit volume in one cycle was obtained using Equation (39):

(39) $$W_{d}=\int_{0}^{T}\frac{Y_{2}}{\omega }{\dot{\varepsilon \left(t\right)}}^{2}dt$$

The average energy lost per unit time, *D*, was expressed as:

(40) $$D=W_{d}/T$$

According to Equation (40), at a known alternating strain ε(t), the energy lost per unit volume and the average energy lost per unit time was determined for different materials. According to the expression and its own solid model, the angular velocity was obtained when the energy consumption of the involute splines was minimal. This facilitated practical applications. The specific calculation procedure is presented in the following example.

Under alternating strain, the energy consumed per unit volume of the involute splines was calculated as:

(41) $$\begin{array}{ll} W_{d} & =\int_{0}^{T}\frac{Y_{2}}{\omega }\varepsilon_{0}{}^{2}\omega^{2}(sin\omega t{)}^{2}dt\\ & =\pi \varepsilon_{0}{}^{2}Y_{2} \end{array}$$

The energy lost per unit time was:

(42) $$\begin{array}{ll} D & =\frac{\pi \varepsilon_{0}{}^{2}Y_{2}}{T}\\ & =\;\frac{1}{2}\varepsilon_{0}{}^{2}\omega Y_{2} \end{array}$$

The solid model of the involute splines was a three-element model with a loss modulus $Y_{2}$ of:

(43) $$Y_{2}=\frac{\left(q_{1}-p_{1}q_{0}\right)\omega }{1+p_{1}^{2}\omega^{2}}$$

$Y_{2}$ was inserted in the equation for energy loss per unit time to obtain:

(44) $$D=\frac{1}{2}\varepsilon_{0}{}^{2}\frac{\left(q_{1}-p_{1}q_{0}\right)\omega^{2}}{1+p_{1}^{2}\omega^{2}}$$

**Figure 7:**
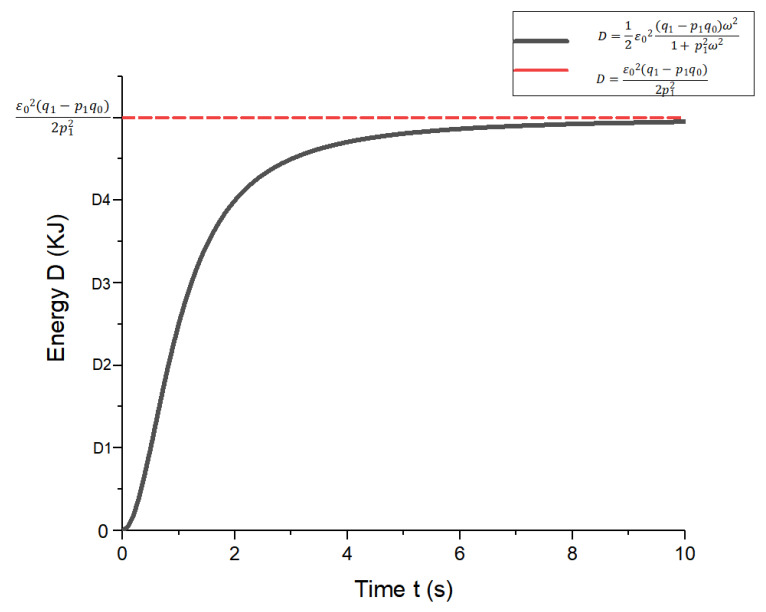
Energy function diagram per unit time loss.

MATLAB was used to graph the function (Figure 7).

From Equation (44), it can be obtained that when ω = 0, involute splines have no energy loss (*D* = 0), when ω→∞ its energy loss for a fixed value. The energy loss of the involute splines was influenced by the angular velocity; the function increased monotonically; greater angular velocity resulted in higher energy loss per unit time. According to Equation (44), we determined the angular velocity when the energy loss was minimum, which resulted in optimal alternating stress to achieve a long lifetime. Periodic engagement forces at different angular velocities, i.e., at different frequencies, caused the internal energy consumption of the involute splines to emit a certain amount of heat, which affected its mechanical properties during operation. Therefore, when designing involute splines in PEEK-based materials, energy loss must be taken into account to ensure stability and accuracy of operation.

The temperature field and surface temperature of the spline sub were calibrated and analyzed, and the working surface temperature of the composite spline was calculated [34]:

(45) $$T=T_{m}+\Delta T_{f}+\Delta T_{c}$$

Each parameter in the formula was calculated as follows:

(46) $$\Delta T_{c}=A\frac{\omega \tau }{1+\tau^{2}\omega^{2}}$$

(47) $$A=\frac{\Pi^{3}F^{2}}{32VcEb_{H}^{2}J}$$

(48) $$\omega =\frac{\Pi^{2}n\rho 1}{60b_{H}}$$

where τ is the delay time, roughly calculated, and the formula for estimating the maximum starting time of the motor is [34]:

(49) $$\tau_{\max}=2\sqrt{P_{e}}+4$$

According to Hertzian contact theory, the following equations were obtained:

(50) $$b_{H}\;={\left(\frac{4F_{t}\rho_{1}}{\pi E}\right)}^{1/2}$$

*ρ_1_* = *R*sin*a* (51)

(52) $$E=\frac{1-V_{1}^{2}}{E1}+\frac{1-V_{2}^{2}}{E2}$$

(53) $$\Delta T_{f}=0.83\frac{\mu W\left|v1-v2\right|}{\left(\sqrt{v1c1\lambda 1V1}+\sqrt{v2c2\lambda 2V2}\right)J\sqrt{b_{H}}}$$

(54) $$T_{m}=T_{0}+0.11W({}^{\circ}C)$$

where *J* is the mechanical equivalent coefficient, and the value of mechanical equivalent was taken as *J* = 4.2 (J/kcal). The determined value of the mechanical equivalent proved that there was a definite quantitative relationship between work and heat, i.e., 1 cal = 4.2 J, or 1 J = 0.24 kcal [34]; *T*_0_ was the temperature of the ambient medium at the periphery of the tooth surface. The maximum temperature of all parts of the aircraft parked in the outside field reached 70 °C; *T*_0_ was set to 80 °C [36]:

The conversion equation of energy consumption to temperature was:

(55) D=cmΔt

Equation (44) was inserted in Equation (55), and the expression for temperature difference △t was obtained as follows:

(56) $$\Delta t=\frac{1}{2}\varepsilon_{0}{}^{2}\frac{\left(q_{1}-p_{1}q_{0}\right)\omega^{2}}{(1+p_{1}^{2}\omega^{2})cm}$$

According to the mathematical equations established in this section, some energy was consumed when the spline sub moved at a certain angular velocity; this energy consumption generated heat, resulting in higher internal temperature of the spline sub. The spline temperature field was calibrated considering the temperature of the tooth face before engagement, the instantaneous internal consumption temperature increase at the engagement point, and the instantaneous friction temperature increase at the engagement point. The method can be used for calculating the spline tooth face temperature of any material. The spline surface temperature of the PEEK-based material was not completely determined, so the tooth surface temperature of the spline sub was further calibrated. Based on the calculated spline sub-working surface temperature T above and the temperature generated by the energy consumption in this section, the total surface temperature of the spline sub-working surface of the PEEK-based material was calculated using Equation (57):

(57) $$T_{q}=T+△t$$

Inserting Equations (45) and (56) into Equation (57), the specific expression of the total surface temperature equation for the spline sub-surface of PEEK-based materials was obtained:

(58) $$T_{q}=T_{m}+\Delta T_{f}+\Delta T_{c}+\frac{1}{2}\varepsilon_{0}{}^{2}\frac{\left(q_{1}-p_{1}q_{0}\right)\omega^{2}}{(1+p_{1}^{2}\omega^{2})cm}$$

The final calculation of the total surface temperature of the spline vice of the PEEK-based material was considered under the influence of viscoelasticity. If the working surface temperature of the spline was less than the allowable continuous working temperature of the material, i.e., $T_{q}<$[△*T*], the PEEK-based involute splines were considered to meet the design requirements, and if $T_{q}>$[△*T*] did not meet the design requirements, it was redesigned.

Equation (58) indicates that the involute splines’ surface temperature was influenced by various factors. The temperature field calibration did not consider the influence of the material, which resulted in a smaller calculated value of the temperature and led to the failure of the designed spline pair before the ideal temperature was reached during operation. This greatly affected the accuracy of temperature calibration and hampered the safety of the transmission system. The stress–strain of an ideal spline pair is constant; therefore, the stress–strain relationship was not given in the actual working conditions leading to ε(t) = 0, and the energy consumption obtained was also constant to 0. Equation (58) was used to calculate the total surface temperature of the involute splines, which was the temperature state of the involute splines of the PEEK-based material under the influence of viscoelasticity. Thus, involute splines using PEEK-based materials can be used to design better matches for actual working conditions, with more accurate and reliable temperature field calibration.

### 2.4. Analysis of Strength Calculation Results

Strength checks were performed for the conditions given in Section 2.1.6 and the basic geometric parameters of the designed involute splines. The values of the parameters used in the calibration process are shown in Table 2, and the calibration results are shown in Table 3.

For the involute spline pair designed in Section 2.1.6, the tooth contact strength, tooth root bending strength, tooth root shear strength, outer spline torsion, bending resistance strength, inner spline shear strength, and spline pair strength all satisfied the strength requirements, allowable temperature range, and wear resistance strength under the cycle number; however, it did not meet the long-term wear-free working conditions. The meanings of all parameters in this work are given in Appendix A.

**Figure 8:**
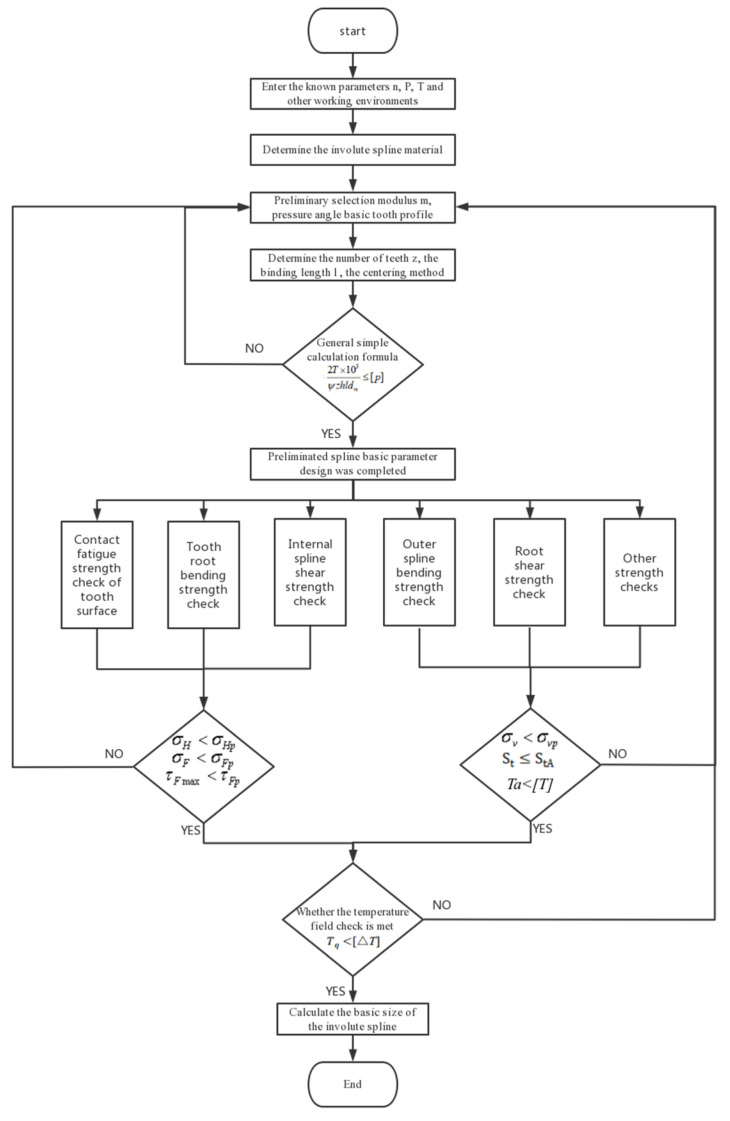
Block diagram of splines design process.

The flow chart of the PEEK-based material involute splines design is shown in Figure 8:

## 3. Contact Characteristics Analysis

In this section, we investigated the effect of geometrical parameters of the designed spline on its contact characteristics. Finite element models were created for the involute splines using PEEK-based materials. The main form of failure of involute splines is crushing of the working surface or excessive wear of the working surface; thus, this section also focused on verifying the strength check results in Section 2.2 only in terms of tooth contact compressive stress distribution and tooth root bending stress distribution, analyzing the tooth contact transient state more precisely, conducting targeted analysis and tooth surface optimization, and evaluating whether the design and check results of PEEK-based material involute splines are feasible for practical applications.

### 3.1. Solid Modeling of Involute Splines

Involute splines developed using PEEK5600CF30 were used to study their operation under a certain torque, and their contact stresses and slip distances were analyzed.

**Figure 9:**
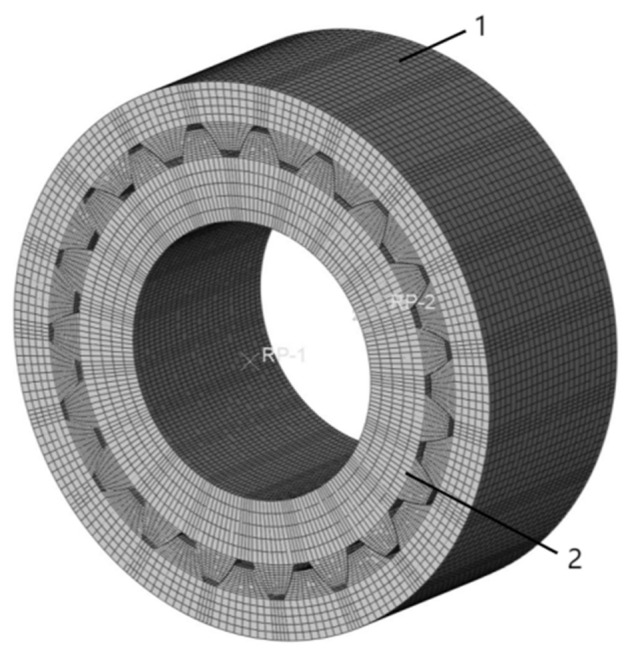
Involute splines assembly model; 1 is Inner Spline and 2 is External Spline.

For example, for the involute splines under the actual working condition in Section 2.1.6, considering the main characteristic parameters of the involute splines engagement in Table 2, MATLAB was used to establish the 3D model of the inner and outer splines. A model was established to obtain the 3D model of the involute splines as shown in Figure 9.

### 3.2. Finite Element Modeling of Involute Splines

The previously created 3D assembly model was saved in an intermediate format file and then imported into the finite element simulation program Abaqus, where finite element models of the internal and external splines were created for analysis and calculation.

The specific steps of the involute splines finite element model building are as follows:

(1) Define the properties of the material: The involute splines materials used were PEEK5600CF30 or PEEK5600LF30 with a density of 1.4 g/cm^3^, modulus of elasticity of 3.5 GPa, Poisson’s ratio of 0.35, and coefficient of friction of 0.27. Due to some unique characteristics of the material and viscoelasticity, it is unlike metal materials. Thus, in the ABAQUS2022 software, we set the elastic force and also defined the viscoelasticity and entered the corresponding shear modulus G1 (value calculation formula in Equation (59)); the internal and external spline materials were the same.

(59) $$G1=\frac{E}{2\left(1+V\right)}$$

where *E* is the modulus of elasticity and *V* is the Poisson’s ratio.

**Figure 10:**
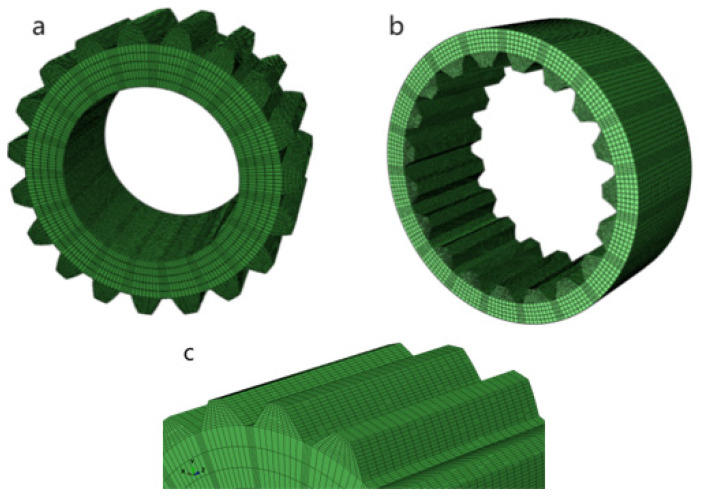
Involute splines finite element mesh model. (**a**) external spline, (**b**) internal spline, (**c**) mesh densification diagram.

(2) Dividing the grid: A hexahedral cell was used for meshing, and its cell type was C3D8R. After preliminary calculations, the contact force area was the two lateral edges of the involute splines, so the two contact edges of the involute splines were densified by the mesh (Figure 10c). When densifying the mesh, the density of the mesh was appropriately reduced in the contact edge area and appropriately increased in the contact area, completing the manual division of the mesh. The involute splines vice model after dividing the mesh is shown in Figure 10. There were 134,976 units and 162,452 nodes.

(3) Create boundary conditions and apply loads: The boundary conditions were set to release rotation and movement in the *Z*-axis direction only. The end face of the inner spline was fixed; a constant torque of 1751 N·m and a constant speed of 6000 rpm were applied to the inner surface of the outer spline, so that the outer spline tooth face was active and the inner spline tooth face was driven. The effect on contact performance of involute splines under this condition was analyzed. There were two analysis steps this time, initial and step 1. The analysis step is dynamic and explicit. After specifying the load, speed, and boundary conditions, the outer surface of the outer spline and the inner surface of the inner spline were selected and subjected to specific contact conditions and constraints before being solved.

**Figure 11:**
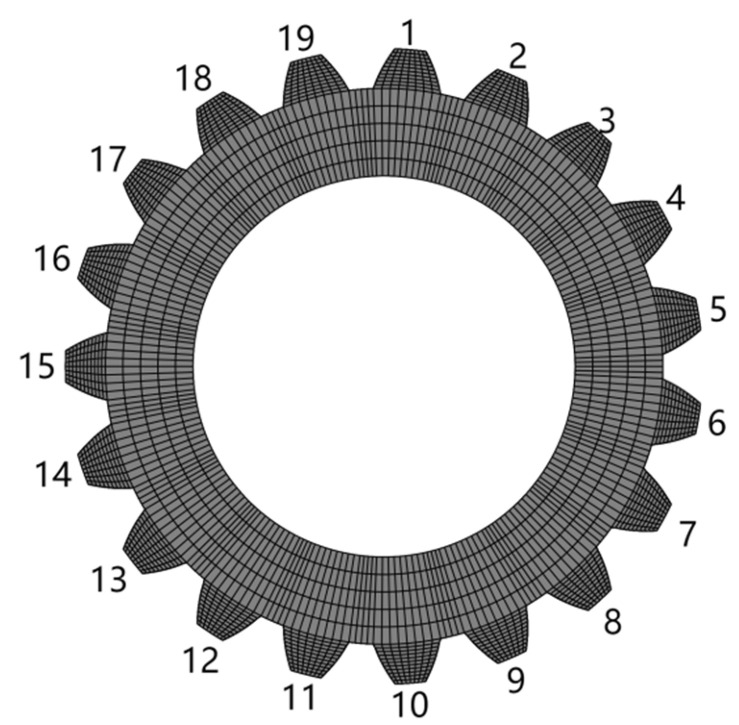
Key tooth numbering diagram.

The key teeth were numbered for ease of study, and the distribution of stress calculation results is represented in the form of a rectangular mapping window (Figure 11).

### 3.3. Simulation Results and Analysis

**Figure 12:**
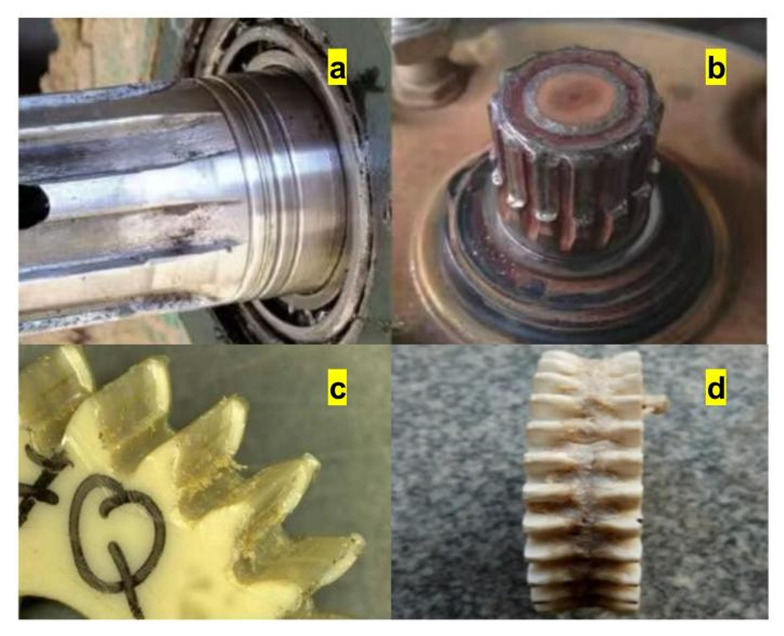
Engineering failure diagram. (**a**,**b**) is Involute splines failure (**c**,**d**) PEEK gear failure.

According to a large number of PEEK polymer composite gear failure forms and engineering failure parts analysis of involute splines vice, as shown in Figure 12, and combined with the existing relevant research status of the preliminary conclusion [3,37], the main failure forms of PEEK-based material involute splines vice were determined to be crushed working surfaces and excessive wear on the working surfaces. These two forms of failure are usually related to the tooth root bending stress and tooth surface contact stress, so in this section, we verified the tooth surface contact compressive stress and tooth root bending stress in the strength calculation results in Section 2 through finite element simulation to further ensure reliability of the proposed PEEK-based material involute splines vice design method.

#### 3.3.1. Tooth Surface Contact Compressive Stresses

**Figure 13:**
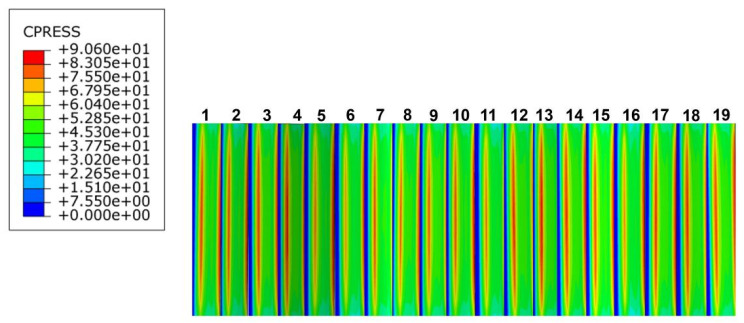
Contact stress cloud diagram.

Based on the above actual working conditions, the finite element model of the PEEK-based material involute splines was simulated, the tooth contact stresses during the engagement of the involute splines were calculated, and the contact stress distribution clouds of the engaged parts of the spline were obtained as shown in Figure 13.

According to Figure 13, the maximum stress of the involute spline pair varied periodically during the movement. The contact stress increased from the root to the top of the tooth, was maximum when it reached tooth surface, and then gradually decreased. Therefore, the maximum contact stress was distributed on the tooth surface of each tooth, and the maximum tooth surface contact stress, as observed in the Figure 13, was *σ_Hmax_* = 90.6 MPa, and in its working process, each tooth face was subjected to a series of contact stresses; the tooth contact stresses are shown in Figure 13.

**Figure 14:**
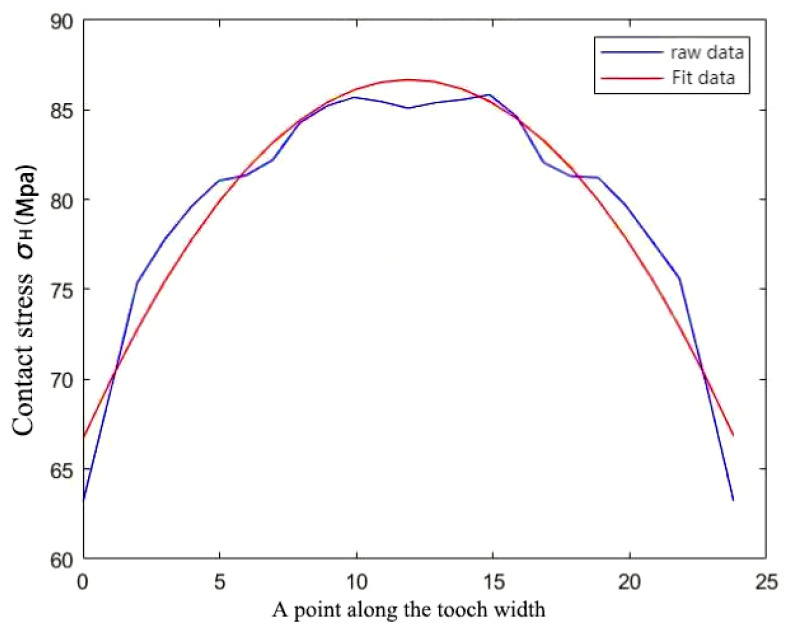
Stress dispersion.

The contact stress on the tooth surface varied periodically, and the contact stress appeared to reach a large peak when entering the engagement and the center of the tooth surface; that is, there was a meshing shock when the involute splines entered the engagement. The peak contact stress at the center of the tooth surface was attributed to the location of the center position along the tooth width direction stress distribution as shown in Figure 14, which was consistent with the contact stress distribution on the tooth surface of the involute splines [38], and the Figure 14 analysis results were reasonable.

The theoretically calculated value of tooth surface contact compressive stress was *σ_H_* = 80.3122 MPa, while the maximum tooth surface contact stress obtained from simulation was *σ_Hmax_* = 90.6 MPa. The theoretical value error rate was 11.3%, which was within the error tolerance. Error is inevitable in the involute splines theory calculations, especially under the condition of friction; therefore, the contact stress distribution could not be accurately obtained. Simulation results obtained using finite element modeling were more accurate, not much different from the contact stress calculation results above, and satisfied the strength check formula. Stress analysis is an extremely important part of the design of involute splines in PEEK-based materials. The finite element method is more convenient than traditional calculation methods and provides a reference for the design of PEEK-based involute splines. It can ensure that the PEEK-based involute splines have adequate tooth contact fatigue strength while meeting the required transmission characteristics.

#### 3.3.2. Tooth Root Bending Stress Distribution

**Figure 15:**
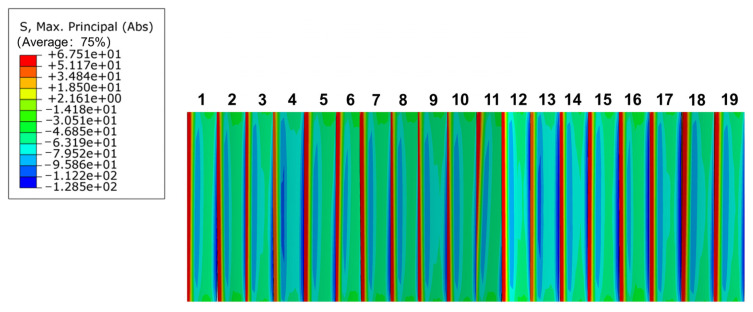
Bending stress distribution diagram of internal and external spline.

The involute splines’ engagement process is full tooth engagement, resulting in its bending stress distribution being on each tooth, so the maximum stress in each tooth is the bending stress at the root of the tooth. While studying its bending stress distribution, the stress concentration area was analyzed. Stress concentration considerably influenced the fatigue life of the member, and the stress concentration caused brittle material fracture and fatigue cracks in brittle and plastic materials. Therefore, whether it is a fatigue problem with brittle or plastic materials, the effect of stress concentration must be considered. With a finite element analysis of the inner and outer splines, bending stress distribution of the inner and outer splines can be easily determined, as shown in Figure 15.

From Figure 15, it is obvious that the bending stress is at its maximum at the root of the inner and outer splines, and the stress first decreases and then increases from the root to the top of the tooth, which conforms to the actual conditions of the involute splines in the meshing transmission process. This phenomenon was caused by a small over-rounding of the tooth root, which increased stress concentration at the root. The root part of involute splines is usually cut or cracked during the machining process, which aggravates the stress concentration phenomenon at the root. For long-term heavy-duty operation of the involute splines, the root part of the tooth is subjected to continuous changes in stress impact, which is most likely to produce fatigue cracks and eventually leads to tooth root fracture; therefore, materials should be selected with due consideration, i.e., the yield stress of PEEK-based materials and the actual operating conditions, among other factors, should be considered to avoid involute splines failure.

**Figure 16:**
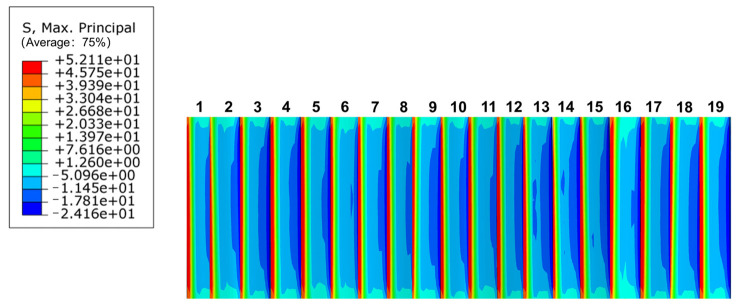
Maximum bending stress distribution of internal spline.

In summary, root bending stress of the PEEK-based spline should be calibrated and analyzed to ensure that the bending strength meets the strength requirements. The maximum bending stress distribution of the external spline, as shown in Figure 16, shows that the bending stress was greatest at the root of the tooth and decreased from the root to the top of the tooth, differing greatly from the bending stress at the root of the tooth. Therefore, the involute splines had a stress concentration at the tooth root. Bending stress at the root of the tooth was $\sigma_{Fmax}\;$= 52.11 MPa. The allowable tooth root bending stress for PEEK-based material involute splines was $\sigma_{Fp}\;$= 329.375 MPa. According to the calibration conditions, $\sigma_{Fmax}<\sigma_{Fp}$. It was within the strength range to ensure that the PEEK-based material’s involute splines transmission characteristics satisfied the requirements and exhibited sufficient tooth root bending fatigue strength.

## 4. Parameter Optimization Design

In Section 2 and Section 3, involute splines for PEEK-based materials were designed based on international standards and other conventional empirical formulas, but the splines that were created were not optimal. Therefore, this chapter complements and refines the PEEK-based material involute spline design method based on Section 2 and Section 3 by comparing and analyzing the selected parameters to determine the optimal solution for designing PEEK-based material involute splines for certain operating conditions. By analyzing and comparing pressure angle, modulus, bond length, and other characteristics, we designed PEEK-based material involute splines with better performance and improved the design rules thereof. It is crucial to improve the strength and service life of PEEK-based material involute splines [38,39].

### 4.1. Analysis of the Effect on Splines Contact Characteristics at Different Pressure Angles

Pressure angle is a critical parameter for designing PEEK-based material involute splines. The shape and size of the tooth profile are determined by the pressure angle. The size of the pressure angle affects the strength of the spline teeth. If the same circumferential force acts on the spline, the positive pressure increases with a large pressure angle, thereby increasing the frictional force; thus, we should consider selecting the most suitable pressure angle. In this section, the spline designed in Section 2.1.6 is considered to explore the effect of different pressure angles on involute splines while keeping other conditions constant.

The pressure angle and modulus of the spline designed herein were 30° and 2.5 mm, respectively. The pressure angles of involute splines according to international standards are 37.5° and 45°, which correspond to modulus ranges of 0.5–10 mm and 0.25–2.5 mm, respectively. The pressure angle and modulus designed herein were thus between the ranges mentioned above. Contact characteristics were then analyzed for the involute spline pair with 37.5° and 45° pressure angles, and the rest of the parameters were kept constant, same as the parameters given in Section 2.1.6. To determine the optimal pressure angle, the contact characteristics of the PEEK-based involute spline pair were analyzed in terms of tooth contact compressive stress distribution, interdental load distribution, and maximum bending stress distribution.

**Figure 17:**
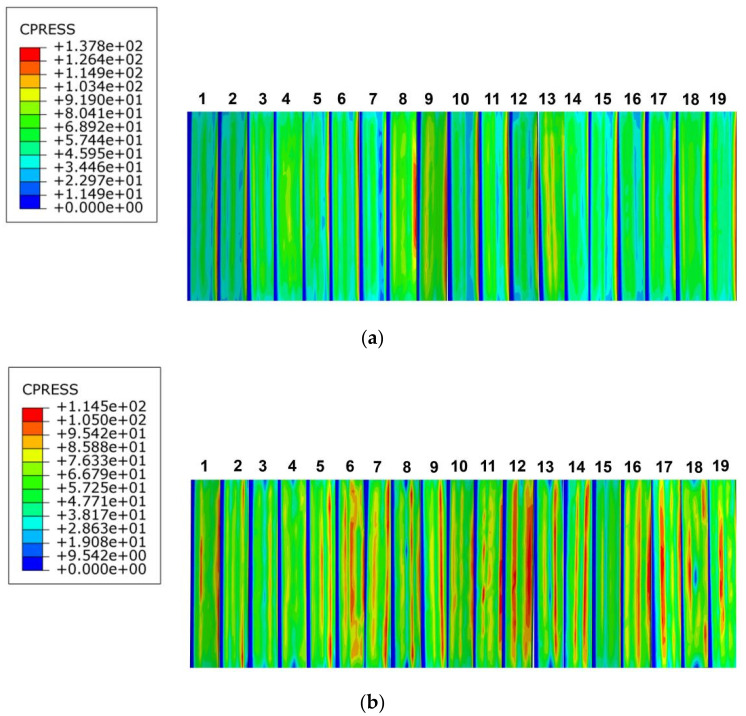
Contact stress distribution of tooth surface with different pressure angle. (**a**) 37.5° pressure angle, (**b**) 45°pressure angle.

The distribution of tooth surface contact compressive stresses in the PEEK-based material involute splines subassemblies at pressure angles of 37.5° and 45° is shown in Figure 17a,b; the contact compressive stresses increase from the tooth root to the tooth top. According to the distribution law of compressive stress in the figure, the maximum contact compressive stress on the tooth surface of the spline pair at a pressure angle of 37.5° was 137.8 MPa, and the maximum stresses were all distributed at the top of the tooth; the contact compressive stress at the tooth surface was relatively small, but it was distributed within a range. The maximum contact compressive stress of the spline on the tooth surface at a pressure angle of 45° was 114.5 MPa, which was slightly lower than the maximum contact compressive stress of the spline at a pressure angle of 37.5°. The distribution of the contact compressive stress on the tooth surface was more uniform, but the maximum stress distribution was more concentrated at the top of the tooth.

**Figure 18:**
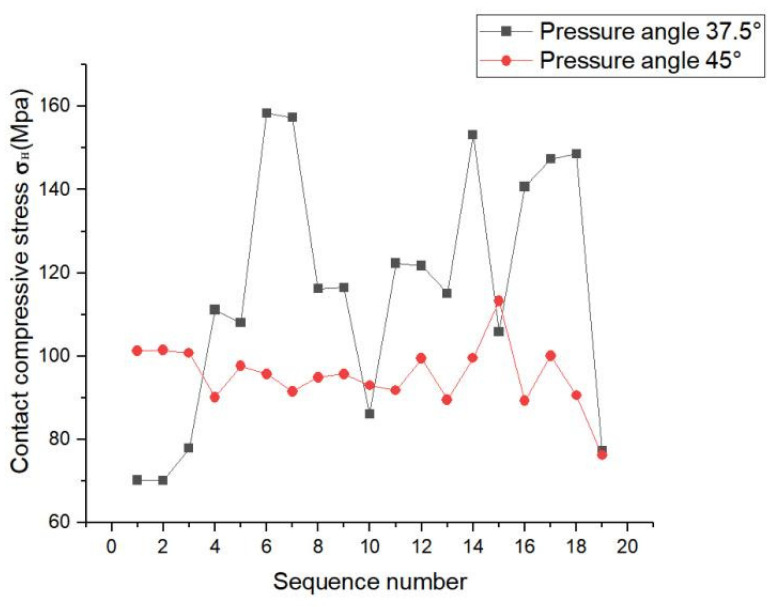
Maximum stress on tooth surface at different pressure angles.

Figure 18 shows the maximum contact stress distribution pattern of each tooth surface. The contact stress at a pressure angle of 37.5° was greater than that at a pressure angle of 45°, so we focused on the compressive stress of the 45°-pressure angle tooth surface.

**Figure 19:**
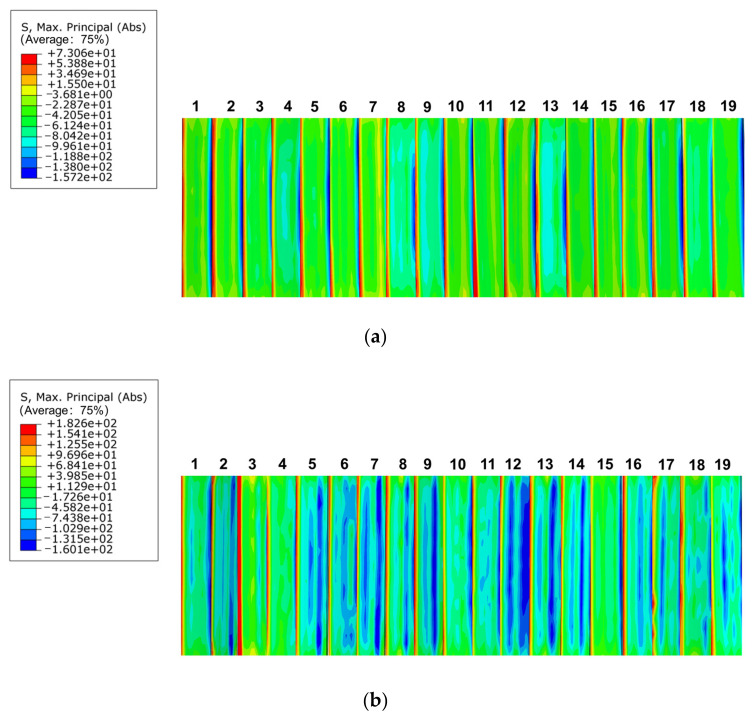
Bending stress distribution at different pressure angles. (**a**) 37.5° pressure angle, (**b**) 45° pressure angle.

As shown in Figure 19a, in the bending stress distribution of the key teeth, at pressure angle of 37.5°, the bending stress distribution was uniform. The stress was smaller at the top part of the teeth, but the bending stress at the root part of the teeth was still relatively large, stress concentration occurred, and the maximum stress was 73.06 MPa. Figure 19b shows that at a pressure angle of 45°, the bending stress distribution was not uniform. Although there was a large bending stress at the root of the tooth, the bending stress at the tooth surface was smaller and more dispersed. The maximum bending stress was 182.6 MPa, which was significantly higher than that of the spline with a pressure angle of 37.5°. However, the overall bending stress of the spline with a pressure angle of 45° was evenly distributed, there was no obvious stress concentration at the root of the tooth, and the maximum bending stress appeared at the top of the tooth.

**Figure 20:**
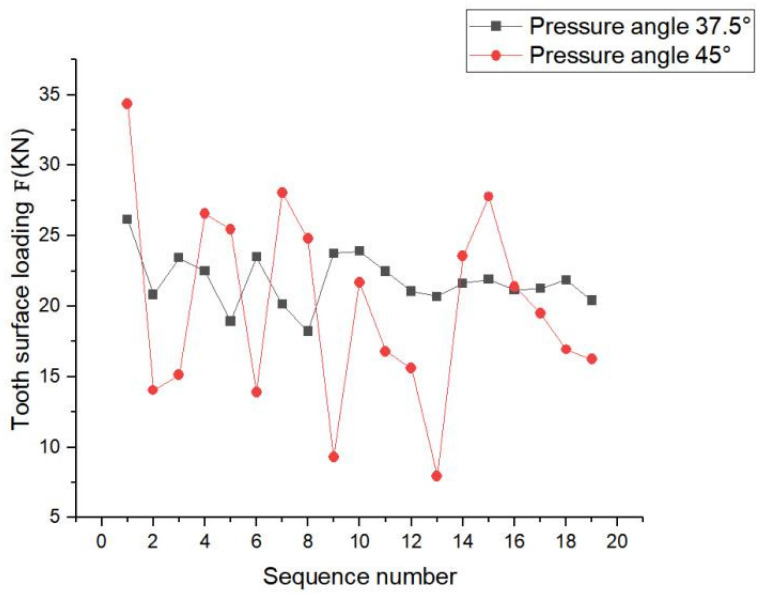
Maximum load distribution of each tooth.

As shown in Figure 20, at pressure angle of 37.5°, the load on the teeth fluctuated to some extent, but the curve was relatively gentle and the load on the teeth was more evenly distributed. At pressure angle of 45°, the load on each tooth of the spline pair fluctuated considerably and was unevenly distributed.

In summary, this phenomenon occurred because with increase in pressure angle, the arc tooth thickness at the top of the tooth decreased, resulting in the tooth becoming sharper and resulting in a lower degree of overlap and making the spline subject to a larger radial force. The spline pair with pressure angle of 45° had better tooth contact pressure distribution and maximum contact pressure distribution on the tooth surface than that with the pressure angle of 37.5°. The load distribution between the teeth of the spline pair with pressure angle of 37.5° was better than the load distribution with a pressure angle of 45°. Based on the above discussion, the following recommendations are made: Other factors should be considered when selecting the pressure angle, and the involute splines pressure angle should be selected based on the required working conditions.

### 4.2. Analysis of the Effect on Splines Contact Characteristics at Different Modulus

The modulus significantly impacts the design of involute splines. The strength of the involute splines is influenced by the magnitude of the modulus. It affects the stability and transmission efficiency. When designing splines, the modulus of different splines should be adjusted according to the working conditions. The effect of spline modulus on contact characteristics was investigated in this study under same working conditions. The findings of this study show that under the same working conditions, splines have a more reasonable modulus range than the general design.

For a constant number of teeth, and because the modulus of the involute splines used herein belonged to the first series, the moduli of 1.25 mm, 2 mm, and 3 mm were selected. We compared and analyzed the contact characteristics of involute splines made using PEEK-based materials under different moduli considering tooth contact compressive stress distribution, inter-tooth load distribution, and maximum bending stress distribution. We derived the results of the influence of the moduli on the spline vices. The findings of this study provide guidance and reference values for practical engineering applications. In this section, the spline designed in Section 2.1.6 was used to explore the effect of different moduli on the involute splines while keeping other conditions constant.

**Figure 21:**
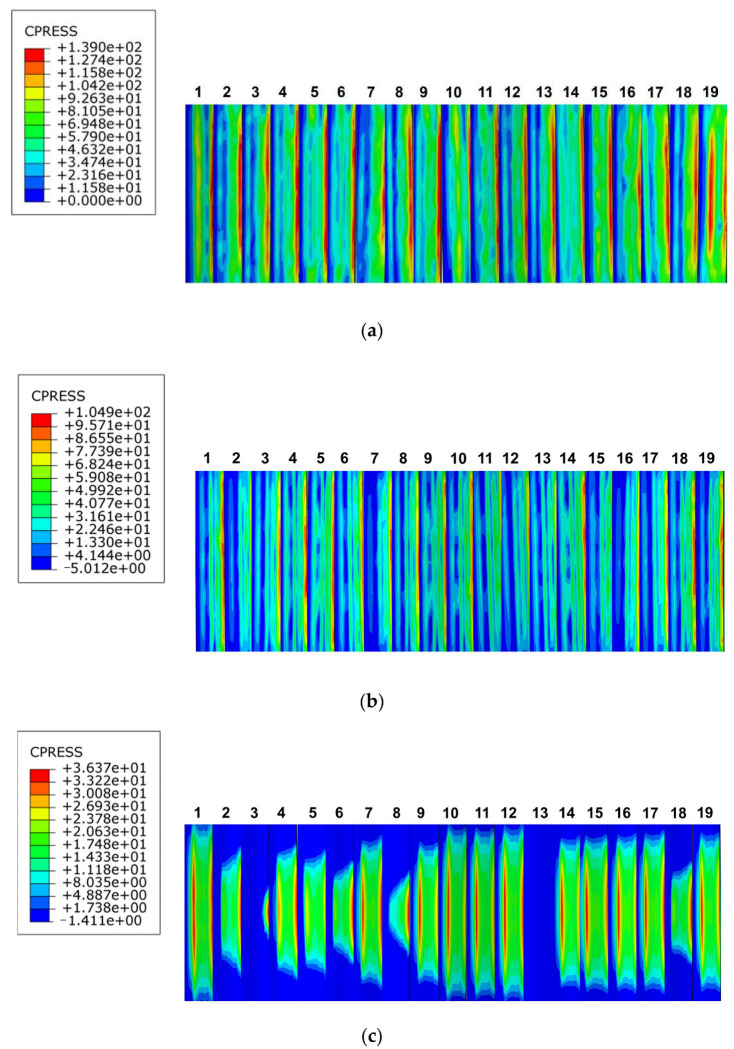
Contact stress distribution of tooth surface with different modulus. (**a**) Modulus is 1.5 mm, (**b**) modulus is 2 mm, (**c**) modulus is 3 mm.

The clouds of the maximum contact compressive stress distribution on the tooth surface of involute splines with different moduli with other circumstances constant are depicted in Figure 21a–c. When the modulus was 1.5 mm, the maximum contact compressive stress on the tooth surface was 139 MPa, and the maximum stress was concentrated at the top of the tooth. When the modulus was 2 mm, the maximum contact compressive stress on the tooth surface was 104.9 MPa. The maximum stress was concentrated at the top of the tooth, but it was not particularly concentrated compared to that at the modulus of 1.5 mm. When the modulus was 3 mm, the maximum contact compressive stress on the tooth surface was 36.37 MPa; the contact compressive stress was not uniformly distributed on the tooth surface, and the concentration phenomenon was not obvious. With increase in modulus, the distribution of tooth contact compressive stress gradually became uneven, and the maximum contact compressive stress on the tooth surface changed from the top surface of the spline sub to the surface of the spline sub. Its maximum contact compressive stress showed a decreasing trend.

**Figure 22:**
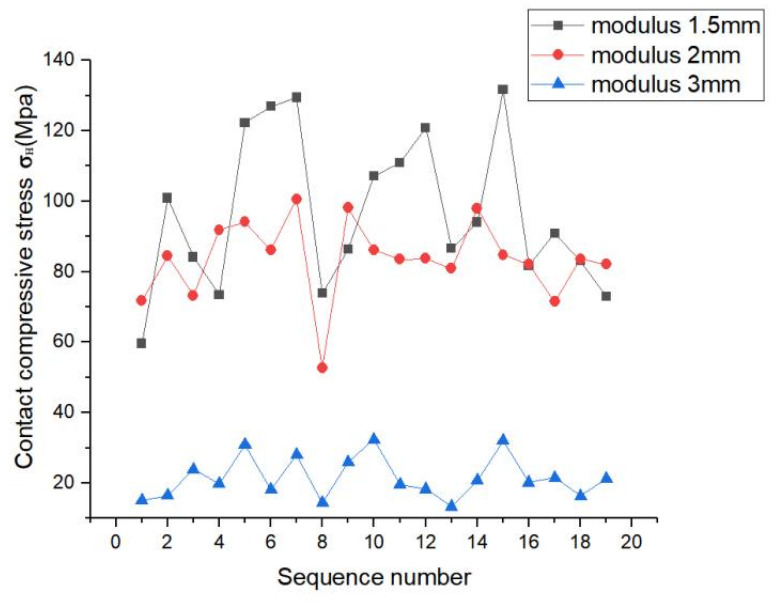
Contact stress line diagram of tooth surface with different modulus.

As shown in the line graph in Figure 22, the contact compressive stress on the tooth surface of the spline decreased with increase in modulus. The contact compressive stress difference of each tooth surface increased when the modulus was 1.5 mm, and the magnitude of the contact stress fluctuation was relatively large. When the modulus was 2 mm, the contact compressive stress on each tooth surface fluctuated, but it was relatively flat; moreover, the amplitude of the contact compressive stress on the tooth surface also decreased. When the modulus was 3 mm, the value of tooth contact compressive stress decreased significantly, the compressive stress on each tooth surface fluctuated less, and the folding line graph was relatively flat. This indicated that with increase in the modulus, the value of the maximum contact compressive stress on the tooth surface decreased, and the stresses on each tooth surface were distributed uniformly.

**Figure 23:**
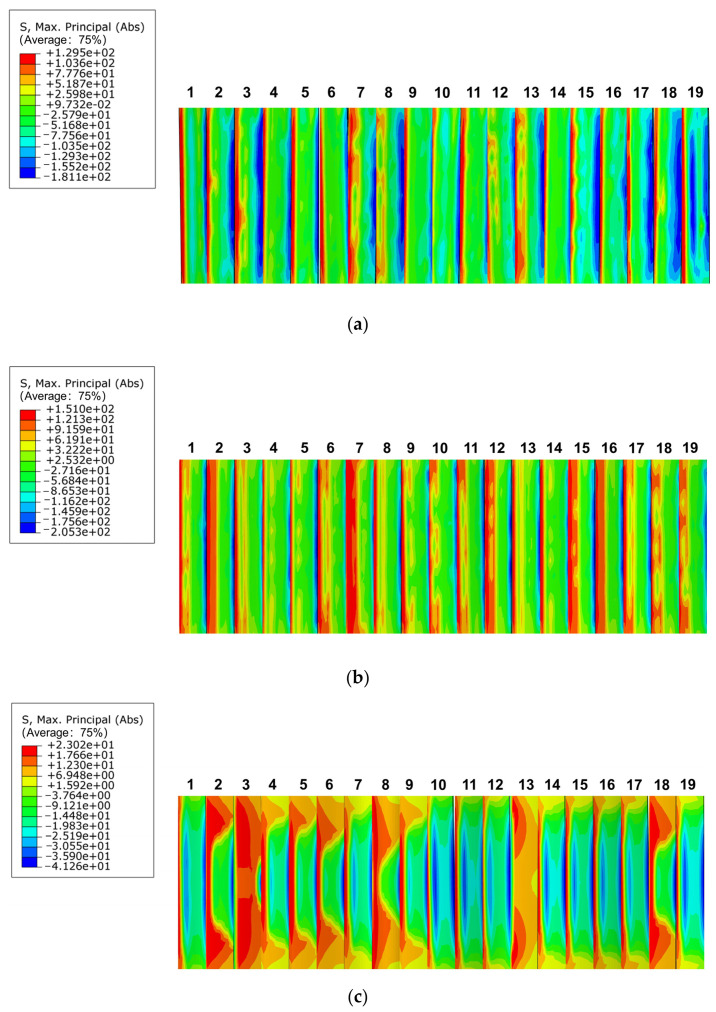
Bending stress distribution of different modulus. (**a**) Modulus is 1.5 mm, (**b**) modulus is 2 mm, (**c**) modulus is 3 mm.

Figure 23 depicts the maximum bending stress distribution clouds on the tooth surface, where Figure 23a–c represent the maximum bending stress clouds for moduli 1.5 mm, 2 mm, and 3 mm, respectively. When the modulus was 1.5 mm, the maximum bending stress was 129.5 MPa, and it was concentrated near the root of the outer spline; however, the stress concentration was not obvious. When the modulus was 2 mm, the maximum stress was 151 MPa, and it was distributed near each tooth heel; however, the stress gradually concentrated. The maximum stress was 23.02 MPa when the modulus was 3 mm, and it was distributed on the tooth root surface. The maximum bending stress of the involute splines was spread from the root to the outer surface, with increase in the modulus, and the stress concentration at the root was obvious at modulus of 3 mm; however, the value of the maximum stress decreased. When the modulus increases gradually from 1.5 mm to 3 mm, the value of the maximum bending stress first increased and then decreased; as a result, the maximum bending stress distribution was relatively obvious because of the influence of the modulus. However, it did not continue to increase with increase in modulus and had to be determined according to the specific working conditions and the requirements of the tooth root modulus to meet the actual maximum bending stress requirements.

**Figure 24:**
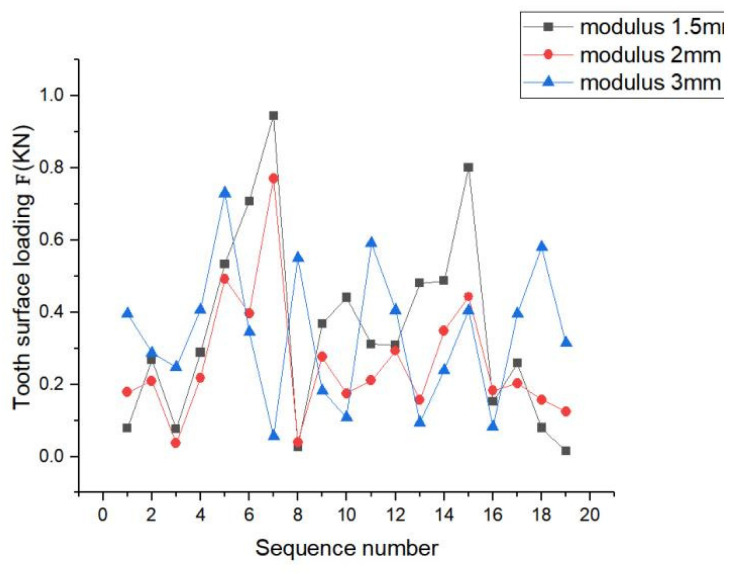
Load distribution with different modulus.

The load distribution for moduli 1.5 mm, 2 mm, and 3 mm is depicted in Figure 24. The peaks of the load were observed on teeth 4 and 7. The load fluctuations of the three moduli on each tooth surface were relatively large, showing a non-uniform trend, and they all had largely similar peaks. The difference between their peaks was relatively small. The three cases did not have considerable differences, and their variations were similar, but the fluctuations of the modulus at 2 mm were relatively flat compared to that at other two moduli; thus, the stability of the load was further adjusted according to the requirements.

In summary, this phenomenon occurred because the modulus increased, which increased the overlap to improve the smoothness as well as the contact fatigue strength. The tooth contact pressure distribution and tooth root bending stress distribution of the spline with modulus of 1.5 mm were better than those of the spline pairs with moduli of 2 mm and 3 mm. The load distribution for the three cases did not differ considerably, and their trends were similar. Based on the above discussion, the following recommendation is given: When selecting the modulus of the involute splines sub-modulus, a smaller modulus should be selected to improve the excellent contact characteristics of the spline.

### 4.3. Analysis of the Effect on Splines Contact Characteristics at Different Engagement Length

The engagement length of involute splines made using PEEK-based materials is a crucial geometric parameter. The load-bearing ability of the involute splines and their other comprehensive properties are influenced by the bond length. Engagement length should not be excess during the design phase because it may result in uneven load distribution along the spline axis. Moreover, increasing the engagement length does not increase the load carrying capacity. Therefore, in this section, the spline designed in Section 2.1.6. is considered to investigate the effect of different engagement lengths on the contact characteristics of the involute splines, keeping other conditions constant.

**Figure 25:**
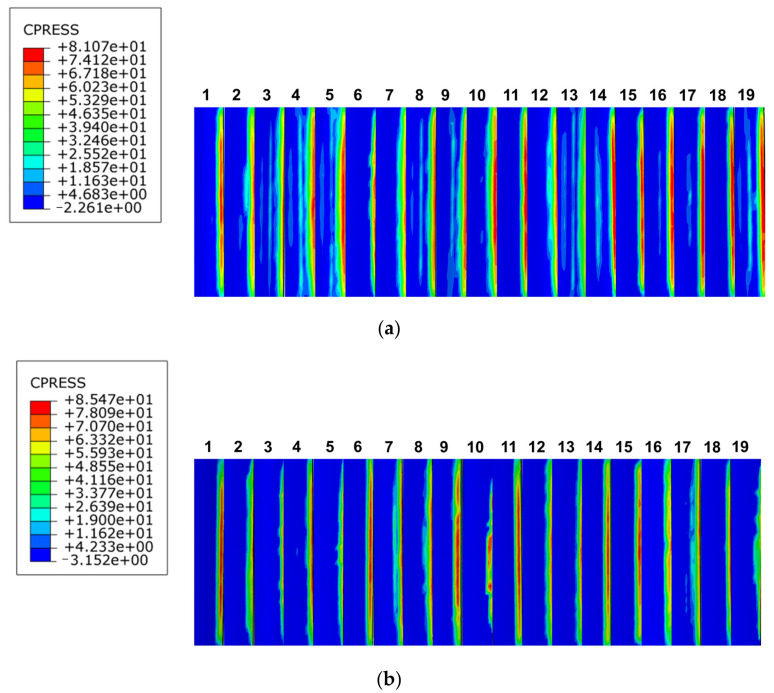
Contact stress distribution of tooth surfaces with different engagement length. (**a**) Engagement length is 30 mm, (**b**) engagement length is 40 mm.

Engagement lengths of 30 mm and 40 mm were used to examine and analyze the effects of spline engagement length on the contact characteristics of the spline pairs from three perspectives, namely, tooth contact pressure distribution (Figure 25), inter-tooth load distribution, and maximum contact pressure distribution. The effects of bond length on the spline pairs were determined. We compared the involute splines design to the original design scheme for determining whether it is reasonable, and then, we made specific recommendations for offering a workable and useful reference for practical engineering applications.

At engagement lengths of 30 mm and 40 mm, the maximum contact stress on the tooth surface was 81.07 MPa and 85.47 MPa, respectively. With increase in engagement length, the maximum contact stress on the tooth surface increased slightly, but this stress did not decrease with further increase in engagement length. The contact stress distribution of involute splines with different engagement lengths is similar, and the contact stress is distributed at the tip of each tooth.

**Figure 26:**
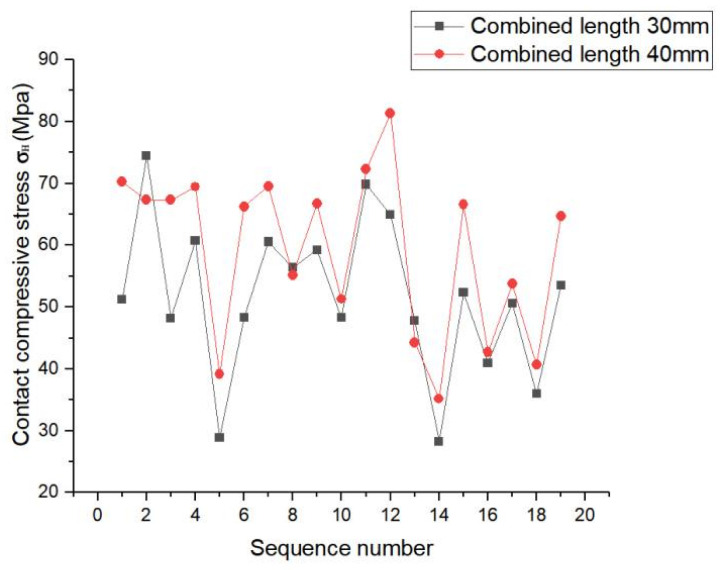
Contact stress line chart of tooth surface with different engagement length.

As shown in Figure 26, the contact stress distribution of each tooth was identical, and each tooth showed similar stress fluctuation trends for each involute splines; however, its fluctuation range was larger. Therefore, tooth contact stress did not significantly improve with increase in bond length. Thus, the following is proposed: engagement length should be selected according to the actual situation and design requirements for obtaining better contact characteristics.

**Figure 27:**
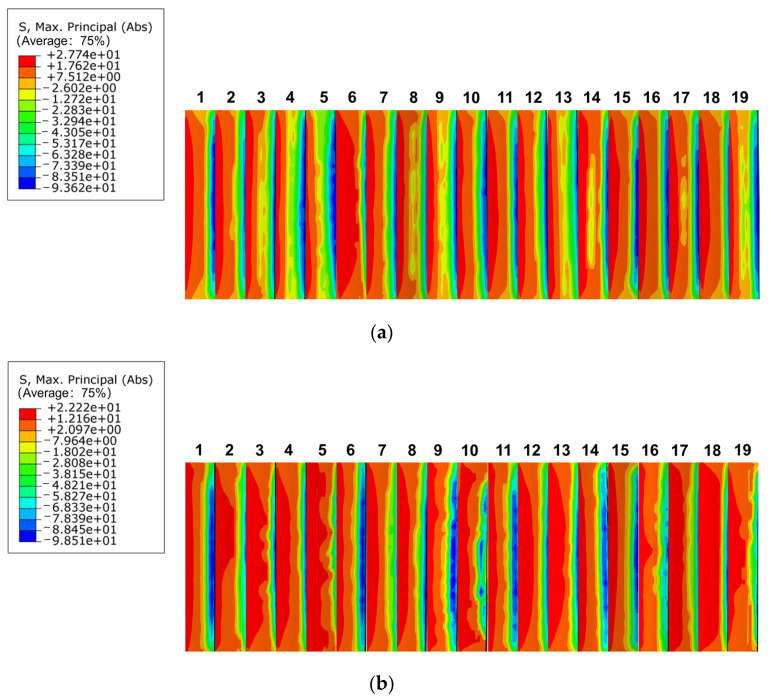
Bending stress distribution of different engagement length. (**a**) Engagement length is 30 mm, (**b**) engagement length is 40 mm.

The maximum bending stress distribution at engagement lengths of 30 mm and 40 mm is depicted in Figure 27a,b, respectively. The stress concentration of the spline was evident at both lengths, and it was concentrated at the tooth root. At engagement lengths of 30 mm and 40 mm, the maximum bending stress was 27.74 MPa and 22.22 MPa, respectively. The stress concentration phenomenon was more severe for engagement lengths >30 mm. As a result, the stress concentration phenomenon improved to some extent with increase in engagement length; however, this does not imply that the stress concentration phenomenon became less obvious with length increase.

**Figure 28:**
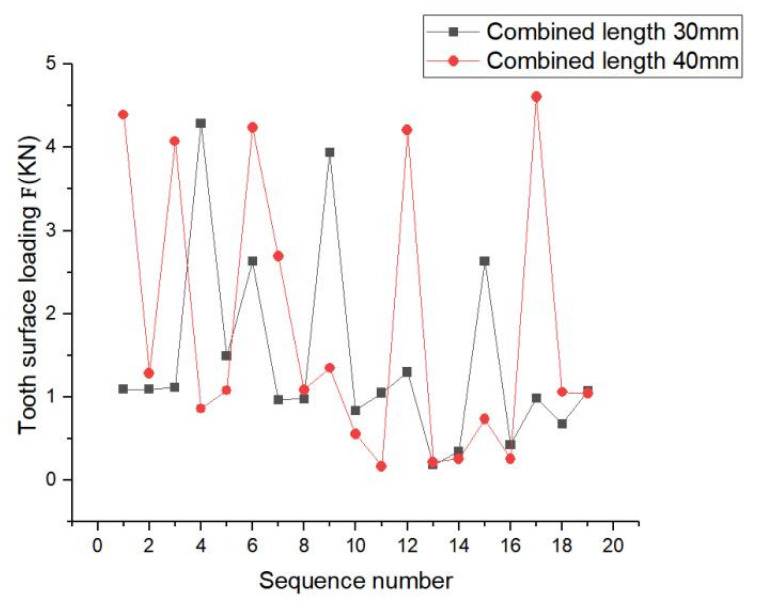
Load distribution for different engagement length.

Figure 28 shows the load distribution of each tooth at engagement lengths of 30 mm and 40 mm. At engagement length of 30 mm, the load in some teeth changed abruptly; however, the overall fluctuation range was small compared to that at engagement length of 40 mm. At engagement length of 40 mm, the load fluctuation range between the teeth was relatively large, which reflected the different and widely varying force conditions between the teeth.

This phenomenon occurred because with increase in engagement length, the key sleeve and spline shaft engaged and transmitted the rated torque, resulting in a longer sliding distance and hence worse in frictional wear. The maximum contact stress distribution and bending stress distribution of the spline tooth surface at engagement length of 40 mm was better than that at engagement length of 30 mm; however, for spline pair with engagement length of 30 mm, the load was better than that for engagement length of 40 mm. Based on the above study, the following recommendation is given: engagement length should be in the range of 30–40 mm according to the actual requirements.

## 5. Conclusions

The design methodology and procedure for PEEK-based material involute splines was established in this study. According to the involute splines design guidelines and design manuals, and considering the influence of viscoelasticity on the design parameters, design and strength check of involute splines parameters for PEEK5600CF30 materials were developed. A finite element model of the involute spline pair of PEEK-based material was created, and the spline pair’s contact characteristics were investigated. The effects of various pressure angles, moduli, and engagement length analysis parameters on spline vice contact characteristics were also investigated, and following conclusions were obtained:

(1) Under given working conditions, according to the PEEK-based material involute splines design theory and strength check method proposed in this study, involute splines were developed using PEEK5600CF30 material. The PEEK5600CF30 material-based involute splines were calibrated for tooth contact strength, tooth root bending strength, tooth root shear strength, outer spline torsion and bending resistance, inner spline shear strength, allowable torque calibration, temperature field analysis, and other strengths. The results satisfied the working requirements and were consistent with the finite element simulation results. This provides a good theoretical basis for the application of involute splines in PEEK-based materials.
(2) The effect of viscoelasticity on the temperature state of the PEEK-based involute splines was more pronounced, and the results were more accurate and reliable when considering the viscoelasticity of the temperature field calibration. This conclusion is crucial for improving the contact characteristics and durability of splines and has a good reference value for engineering practice.
(3) Based on the working conditions in 2.1.6, finite element analysis was performed for the designed splines. Under the condition that other parameters remained unchanged, the involute splines showed better tooth surface compressive stress at a pressure angle of 45° than that at 37.5°; also, the amplitude of its tooth surface contact compressive stress distribution decreased and was more uniform. The stress concentration phenomenon was obvious in the load fluctuation range. Comparing the analysis of involute splines with different moduli, we determined that with increase in modulus, the tooth contact compressive stress became uneven, the maximum contact compressive stress showed a decreasing trend, the value of the maximum stress increased and then decreased, and the load changes were approximately the same. Comparing the analysis of involute splines with different bond lengths, we determined that the tooth contact compressive stress trend remained almost unchanged with increase in bond length, and the load fluctuation range increased with increase in length, and the stress concentration phenomenon improved to some extent.
(4) The bearing capacity of involute spline couplings can be improved by increasing the pressure angle. The appropriate modulus selection can balance the strength and size requirements of the involute spline couplings. Increasing the engagement length of involute spline couplings can increase the contact area and enhance the connection effect of involute spline couplings. It is recommended to increase the engagement length of the involute spline coupling appropriately. In practical applications, it is necessary to select the appropriate parameters, such as pressure angle, modulus, and engagement length, according to the operating environment and bearing requirements of the involute spline couplings to meet the performance requirements of the involute spline couplings. Future development can further study the optimization of involute spline couplings materials. Through the improvement and upgrading of materials, it can improve the wear resistance, corrosion resistance, fatigue resistance, and other key properties of the involute spline couplings so as to improve the overall performance of the involute spline couplings, which can effectively improve the performance of the transmission system. This has very practical significance.

## Figures and Tables

**Table 1:** Basic dimensions of involute splines.

Symbol	Variable	Value
*z*	Number of teeth	19
*m*	Modulus (mm)	2.5
*α_D_*	Pressure angle (°)	30
*l*	Engagement length (mm)	23.75
	Tooth profile	The root of the tooth is round
*D*	Reference diameter (mm)	47.5

**Table 2:** Table of calculated parameters taking values.

Symbol Description	Takes Values	Symbol Description	Takes Values
Coefficient of safety for tooth contact strength *S_H_*	1.35	Mechanical equivalent of heat *J* (J/Kcal)	4.2
Utilization factor *K*_1_	1	Poisson’s ratio *V*	0.35
Tooth side clearance coefficient $K_{2}$	1	Rockwell hardness *C*	123
Distribution coefficient $K_{3}$	1	Friction coefficient *μ*	0.27 (μ)
Axial load factor $K_{4}$	1.6	Melting point *T* (°C)	343
Conversion factor *K*	0.3	Thermal conductivity *λ* (W/(m·k))	0.3
Lewisian Factor *Y*	1.5	Modulus of elasticity *E* (GPa)	3.5
Load distribution Coefficient $K_{m}$	1	Density *ν* (g/${\mathrm{cm}}^{3}$)	1.4
Uneven factor of load between teeth ψ	0.75	Safety factor *s*	1
Specific heat *c* ((KJ/(Kg·°C))	1340		

**Table 3:** Calculation results of involute splines sub.

Symbol Description	Takes Values	Symbol Description	Takes Values
Contact compressive stress on the tooth surface $\sigma_{H}$ (MPa)	80.31	Allowable shear stress of tooth root $\tau_{Fp}$ (MPa)	164.69
Allowable compressive stress of tooth surface $\sigma_{Hp}\;$(MPa)	243.98	Equivalent stress $\sigma_{v}$ (MPa)	181.47
Tooth root bending stress $\sigma_{F}$ (MPa)	1.79	Allowable equivalent stress $\sigma_{vp}$ (MPa)	329.38
Permissible bending stress of tooth root $\sigma_{Fp}$ (MPa)	329.38	Allowable compressive stress of tooth surface $\sigma_{Hp1}$ (MPa)	95.00
Tooth root shear stress $\tau_{Fmax}$ (MPa)	153.80	Allowable compressive stress of tooth surface $\sigma_{Hp2}$ (MPa)	36.82
Yield strength *σ*_0.2_ (MPa)	527.00	Tooth root torsional shear stress $\tau_{tn}$ (MPa)	99.13
Break-away stress $S_{1}$ (MPa)	19.67	Maximum torque *T_a_* (N/m)	1750.83
Tensile tooth bending stress $S_{2}$ (MPa)	28.54	Allowable torque [*T*] (N/m)	16,235
Circumferential stress $S_{3}$ (MPa)	0.02	Temperature before engagement *T_m_* (°C)	86.80
Allowable stress $S_{tA}$ (MPa)	917.70	Instantaneous internal consumption temperature rise △*T_c_* (°C)	6.00 × 10^−5^
Total tensile stress $S_{t}$ (MPa)	48.23	Instantaneous frictional temperature rise △*T_f_* (°C)	10.85
Surface pressure for Hertzian stress $S_{c}$ (MPa)	55.95	Total surface temperature $T_{q}$(°C)	97.65

## Data Availability

Data are contained within the article.

## Appendix A

Interpretation of parameters required for involute spline pair analysis

*b* is involute splines tooth root height (mm).

$b_{H}$ is hertz contact zone half width (mm).

*c* is specific heat capacity of material.

C is involute splines tooth top height (mm).

*C_V_* is action backlash (mm).

*d* is pitch wire (mm).

$d_{1}$ is involute splines pitch diameter (mm).

*d_h_* is involute splines action diameter (mm).

$d_{n}$ is involute splines inner spline root diameter (mm).

$d_{so}$ is spline coupling coupling speed (m/s).

$d_{x}$ is involute splines inner spline outer diameter (mm).

*D* is involute splines indexing circle diameter (mm).

*D_q_* is energy lost (J).

$D_{B}$ is involute splines shaft outer diameter (mm).

*D_ee_* is involute splines outer spline large diameter (mm).

*D_ie_* is involute splines outer spline path (mm).

*D_ii_* is involute splines inner spline path (mm).

$D_{l}$ is involute splines shaft inner diameter (mm).

*D_m_* is involute splines average circle diameter (mm).

*e*_0_ is amount of displacement (mm).

*E* is the elastic modulus of the material.

*f* is involute splines tooth shape is high (mm).

*F* is finale force (KN).

*F_t_* is nominal tangential force (KN).

*g* is minimum root height of involute splines (mm).

*h* is full tooth height (mm).

$h_{f}^{*}$ is root height factor.

*h_w_* is working tooth height (mm).

*J* is thermal work equivalent coefficient.

*K* is conversion factor.

*K*_1_ is use coefficients.

*K*_2_ is tooth side clearance coefficient.

*K*_3_ is distribution factor.

*K*_4_ is axial bias factor.

$K_{m}$ is load distribution factor.

*l* is involute splines bonding length (mm).

*m* is involute splines modulus (mm).

*M_b_* is bending moment (N·m).

*n* is involute splines speed (r/min).

*R* is involute splines indexing circle radius (mm).

*S* is safety factor.

*S_F_* is calculation of the safety factor of flexural strength.

*S_Fn_* is thick chords (mm).

*S_H_* is calculation of safety factor for tooth surface contact strength.

$t_{w}$ is involute splines inner spline wall thickness (mm).

*T* is input torque (N·m).

*Tm* is the tooth surface enters the temperature before meshing (°C).

$T_{q}$ is total surface temperature (°C).

*P* is input power (kW).

*Pe* is motor power rating (kW).

*v* is material density.

$V_{i}$ is involute splines contact point speed (m/s); i = 1, 2 (1—outer spline and 2—inner spline)

*W* is unit tooth width upper meshing force (N/mm).

*Y* is Lewis form factor.

*z* is number of involute splines teeth.

*α* is involute splines toothed angle (°).

$\alpha_{D}$ is involute splines standard pressure angle (°).

*α_tn_* is stress concentration coefficient.

*η* is viscosity coefficient.

$\dot{\varepsilon }$ is strain rate.

*λ* is thermal conductivity of materials.

*σ_b_* is the tensile strength of the material (MPa).

*σ_F_* is tooth root bending stress (MPa).

[*σ_F_*] is allowable root bending stress (MPa).

*σ_Fa_* is bending stress (MPa).

*σ_H_* is tooth surface compressive stress (MPa).

[*σ_H_*] is compressive stress is allowed on the tooth surface (MPa).

[*σ_Hi_*] is allowable compressive stress for tooth surface wear (MPa).; i = 1, 2 (1—outer spline and 2—inner spline)

*σ_s_* is the yield limit of the material (MPa).

*σ_V_* is equivalent stress (MPa).

[*σ_V_*] is permissible equivalent stress (MPa).

*σ*_0.2_ is the yield strength of the material (MPa).

*σ°* is material ultimate stress (MPa).

*τ* is motor delay time (s).

[*τ_F_*] is shear stress is allowed (MPa).

*τ_tn_* is shear stress (MPa).

*τ_Fmax_* is maximum shear stress at the root of the tooth (MPa).

*μ* is material coefficient of friction.

*ρ* is involute splines tooth root fillet radius (mm).

*Ψ* is uneven factor of load between teeth

*ω* is angular velocity (rad/s).

△*t* is energy consumption conversion temperature (°C).

△*Tc* is instantaneous internal consumption temperature rise of the meshing point (°C).

△*T^f^* is the instantaneous friction temperature rise of the meshing point (°C).

[△*T*] is continuous use temperature (°C).

## Appendix B

**Table A1:** PEEK5600CF30 material performance table.

Project	Unit	PEEK5600CF30
Tensile strength (23 °C)	MPa	230
Tensile modulus (23 °C)	GPa	23.8
Tensile elongation (23 °C)	%	1.5
Bending strength (23 °C)	MPa	360
Flexural modulus (23 °C)	GPa	22.1
Compressive strength (23 °C)	MPa	280
Tensile strength	MPa	100
Lzod Impact strength (no notch)	KJ/m^2^	41
Poisson’s ratio		0.35
Rockwell hardness	HRR	123
Coefficient of friction	μ	0.27
Melting index	g/10min	3.5
Melting point	°C	343
Heat deflection temperature	°C	325
Continuous use temperature	°C	260
Thermal conductivity	W/(m·k)	0.3
Elastic modulus	GPa	3.5
Density	g/(cm)^3^	1.4
Than hot melt	KJ/(Kg°C)	1340
